# Sentinel monitoring for resistance to Bt toxins in European corn borer (Lepidoptera: Crambidae) in Canada

**DOI:** 10.1093/jee/toag077

**Published:** 2026-04-23

**Authors:** Jocelyn L Smith, Yasmine Farhan, Jason Wells, Caitlin Congdon, Julien Saguez, Holly Byker, John Gavloski, Josée Kelly, Galen Dively

**Affiliations:** School of Environmental Sciences, University of Guelph Ridgetown Campus, Ridgetown, ON, Canada; School of Environmental Sciences, University of Guelph Ridgetown Campus, Ridgetown, ON, Canada; New Brunswick Department of Agriculture, Aquaculture and Fisheries, Sussex, NB, Canada; Perennia Food and Agriculture Corporation, Kentville, NS, Canada; CÉROM, Centre de Recherche sur les Grains Inc, Saint-Mathieu-de-Beloeil, QC, Canada; Ontario Crops Research Centre—Winchester, University of Guelph, Winchester, ON, Canada; Manitoba Agriculture, Carman, MB, Canada; School of Environmental Sciences, University of Guelph Ridgetown Campus, Ridgetown, ON, Canada; Department of Entomology, University of Maryland, College Park, MD, USA

**Keywords:** European corn borer, resistance, *Bacillus thuringiensis*, Bt corn, resistance monitoring

## Abstract

Transgenic corn *Zea mays* (L.) producing *Bacillus thuringiensis* insecticidal toxins (Bt corn) has successfully controlled the European corn borer *Ostrinia nubilalis* (Lepidoptera: Crambidae), one of the most destructive corn pests in North America, for over 25 years. However, field-evolved resistance to Cry1Fa toxin was first documented in Nova Scotia, Canada, in 2018, with subsequent detections in Quebec and Manitoba. Before these discoveries, no resistance had been reported in *O. nubilalis* populations to Bt toxins, despite ongoing resistance monitoring mandated by regulatory agencies. Sentinel plots monitored in Canada during 2019 to 2023 reveal the first cases of resistance in *O. nubilalis* populations to Cry1Ab and Cry1A.105 toxins. Laboratory bioassays of field-derived populations corroborate these findings. This study serves as a case study demonstrating the value of sentinel plots for detecting field-evolved resistance and providing critical insights into regional resistance dynamics. Enhanced surveillance, particularly in regions with low Bt corn acreage, is essential to mitigate the spread of resistance and preserve Bt corn technology. This study underscores the need for robust and integrated monitoring frameworks to sustain the efficacy of Bt crops and prevent the resurgence of a historically significant corn pest.

## Introduction

Genetically modified corn, *Zea mays* (L.), expressing insecticidal toxins from *Bacillus thuringiensis* (Bt corn), was developed to control European corn borer, *Ostrinia nubilalis* Hübner (Lepidoptera: Crambidae), a major corn pest since its introduction to North America in the early 1900s (Crawford and McLaine 1926, Caffrey and Worthley 1927, Mason et al. 2018). After more than 25 years of successful management of *O. nubilalis* with this technology, significant shifts in the effectiveness of this ground-breaking pest management system have started to occur. The first instance of field-evolved resistance to the Cry1Fa Bt toxin produced by the transgenic event DAS-1507 (co-developed by Dow AgroSciences LLC, Indianapolis, IN and Pioneer Hi-Bred International, Johnston, IA) was reported in *O. nubilalis* populations in Nova Scotia (NS), Canada, in 2018 (Smith et al. 2019). In 2019 and 2020, additional Cry1Fa-resistant *O. nubilalis* strains were detected in Quebec (QC) and Manitoba (MB), Canada, respectively (Smith and Farhan 2023). Before the Canadian detections, no decrease in *O. nubilalis* susceptibility had been reported to any of the commercially available Bt toxins (Cry1Ab (event MON810, Monsanto Company, St. Louis, MO), Cry1A.105 + Cry2Ab (event MON89034, Monsanto Company), or Cry1Fa (Siegfried et al. 2007, Thieme et al. 2018, Tabashnik and Carrière 2019, García et al. 2023).

With the approval of Bt corn commercialization in 1996, the United States Environmental Protection Agency (USEPA) and the Canadian Food Inspection Agency (CFIA) required registrants to conduct resistance monitoring of target pest populations (CFIA 1996, 1997, USEPA 2001, Macdonald and Yarrow 2003). Larvae are collected annually throughout major Bt corn growing regions in Canada and the United States and tested using an artificial diet bioassay involving dilutions of purified Bt toxin solutions to detect shifts in Bt susceptibility over time, relative to a susceptible laboratory strain (Siegfried et al. 2007, Tabashnik et al. 2008, Tabashnik et al. 2009). Before 2018, resistance monitoring by registrants had not detected any evidence of field-evolved resistance in *O. nubilalis* populations to Bt toxins (USEPA 2024).

Several limitations to the diet-overlay bioassay approach have been identified in recent years. First, field collections of larvae have historically been made from regions with large corn acreage and high Bt-corn adoption rates, likely based on the assumption that the risk of resistance evolution would be greatest in these regions. Yet, recent discoveries of *O. nubilalis* Bt resistance in Canada and the United States have been in areas of relatively low corn acreage and short growing seasons where previous resistance monitoring was not conducted (Smith et al. 2019, Fisher et al. 2025). Secondly, efforts to collect sufficient samples of larvae for bioassay testing have been challenging because *O. nubilalis* populations are greatly suppressed in major corn-growing regions due to Bt corn adoption (Hutchison et al. 2010, Dively et al. 2018). This has required rearing laboratory colonies from small samples of field-collected *O. nubilalis* to provide enough individuals for testing, which has likely eliminated many resistant genotypes due to fitness costs (Crespo et al. 2010, Pereira et al. 2011). Furthermore, access to proprietary Bt proteins has been limited in some cases, and there are significant costs in time and labor required for resistance monitoring programs. With the long-term success of Bt corn technology, these factors have contributed to increasingly less robust *O. nubilalis* resistance monitoring over the lifespan of commercialized Bt corn in North America. The discovery of Bt corn resistance in eastern Canada and the United States has highlighted the need for greater surveillance of *O. nubilalis* populations to mitigate the risk of Bt resistance spreading in North America and preventing the resurgence of a major corn pest.

An alternative approach to monitor resistance is the use of sentinel plots of Bt corn planted alongside their non-Bt isogenic cultivars to compare injury caused by target pests (Venette et al. 2000, Dively et al. 2016). This in-field screening method has proven effective for detecting resistance in populations of corn earworm *Helicoverpa zea* Boddie (Lepidoptera: Noctuidae) to Cry1Ab, Cry1A.105, and Cry2Ab in sweet corn, and is currently used by a large-scale network of researchers in the United States and Canada to document increasing injury and field-evolved resistance over time since 2017 (Dively et al. 2020, 2023). More recently, industry providers of the Bt corn technology, working through the Agricultural Biotechnology Stewardship Technical Committee (ABSTC), have initiated a similar sentinel network using isogenic Bt and non-Bt field corn plots in the southern United States. Likewise, since the detection of Cry1Fa resistance in NS in 2018, existing sweet corn sentinel sites in Canada and the United States have been supplemented with field corn hybrids to increase resistance monitoring efforts for *O. nubilalis* during 2019 to 2023. Here, we report on (i) sentinel plot results documenting the first cases of field injury by *O. nubilalis* populations to Cry1Ab and Cry1A.105 corn in Canada, and (ii) laboratory results from continued testing for Bt resistance in field-derived populations of *O. nubilalis* using diet-overlay and tissue bioassay methods. We provide evidence that sentinel plot monitoring is an effective method for detecting shifts in *O. nubilalis* susceptibility to Bt toxins.

## Materials and Methods

### Sentinel Sites

**Fig. 1.**
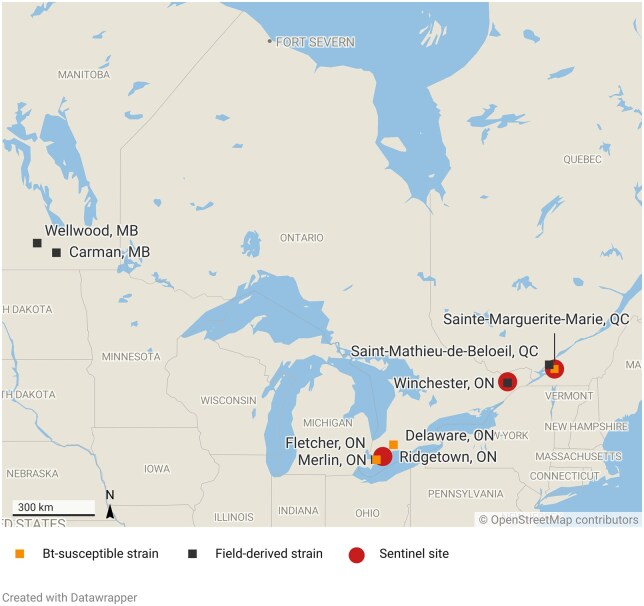
Locations of sentinel sites (circles) and where field-derived strains were collected (black squares) for *Ostrinia nubilalis* Bt resistance monitoring in Ontario (ON), Quebec (QC), and Manitoba (MB), Canada, in 2019 to 2023. Orange squares indicate the collection location of Bt-susceptible laboratory strains.

**Fig. 2.**
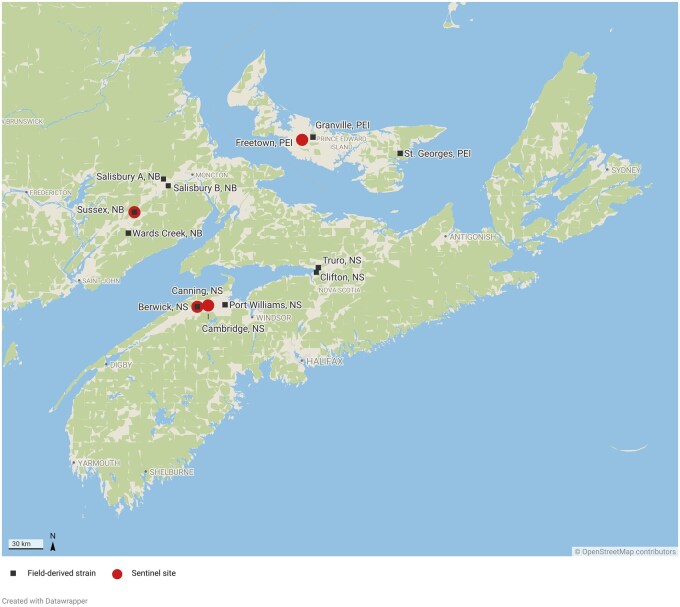
Locations of sentinel sites (circles) and where field-derived strains were collected (squares) for *Ostrinia nubilalis* Bt resistance monitoring in Nova Scotia (NS), New Brunswick (NB), and Prince Edward Island (PEI), Canada, in 2020 to 2023.

Plots of Bt and non-Bt corn were planted in different years at six Canadian sites (Ridgetown, Ontario (ON), 2019 to 2023, Winchester, ON, 2020 to 2023; Saint-Mathieu-de-Beloeil, QC, 2020 to 2023; Sussex, New Brunswick (NB), 2021 to 2022; Berwick/Cambridge, NS, 2020 to 2022; and Freetown, Prince Edward Island (PEI), 2021) (Figs 1 and 2; see details in Supplementary Table S1). At each site, adjacent plots of the following five sweet corn cultivars were planted and sampled according to the methods described by Dively et al. (2020): (i) ‘Attribute BC0805’ producing Cry1Ab, (ii) ‘Attribute II Remedy’ producing Cry1Ab and Vip3A, and (iii) their non-Bt isoline ‘Providence’ (Syngenta Seeds); (iv) ‘Performance Series Obsession II’ producing Cry1A.105 + Cry2Ab, and (v) its non-Bt isoline ‘Obsession I’ (Bayer-Seminis Seeds). In 2020 and 2021, plots of a commercially available grain corn hybrid producing Cry1Fa; hereinafter referred to as Hybrid D (Cry1Fa; 72 comparative relative maturity [CRM]) (Pioneer Hi-Bred Production Co. Chatham, ON) were added to the sentinel sites. In 2022 and 2023, additional plots of grain corn cultivars producing Cry1Fa; hereinafter referred to as Hybrid B (Cry1Fa; 103 CRM), Hybrid C (Cry1Fa and Cry1Ab; 82 CRM), and Hybrid A (non-Bt; 80 CRM) (Pioneer Hi-Bred Production Co., Chatham, ON) were added at all sentinel sites except Cambridge, NS. All plots were planted with pure stands of the cultivar, without integrated non-Bt refuge. Planting configuration was confirmed by qualitative assessment of Bt toxin production in leaf tissue from five randomly selected plants from the central two rows of each plot using EnviroLogix QuickStix (EnviroLogix Inc., Portland, ME). Planting dates were determined based on local agronomic conditions, and all cultivars were planted on the same date within a site (Supplementary Table S1). Plots of each cultivar were 4 to 15 rows wide with 0.76 m row spacing and 15 to 40 m in length (Supplementary Table S1). Corn was planted, fertilized, and managed for weeds using standard agronomic practices specific to each region. No foliar insecticide was applied to any plot, and none of the sites were irrigated.

### Evaluation of *Ostrinia nubilalis* Injury

#### Ear Sampling

At the sentinel sites, kernel injury caused by *O. nubilalis* and other lepidopteran pests was assessed in each Bt and non-Bt sweet corn cultivar at fresh-market maturity, 21 to 25 d after the onset of silking, as described in Dively et al. (2020, 2023). Grain corn ears were examined during the kernel milk (R4) to dough (R5) stages (Abendroth et al. 2011). The number of ears examined ranged from 10 to 200 per cultivar. In general, larger samples were taken in the Bt cultivars to increase the chances of detecting *O. nubilalis* injury and survival. Primary ears on plants from the two central rows of each plot were husked and examined for insect injury to the kernels and ear shank. The severity of injury was visually estimated as the area (cm^2^) of consumed kernels using a transparent square cm grid overlay. The identification, instar, and number of larvae observed in each ear were recorded. At the Sussex, NB site in 2023, only a small number of ears (i.e., 10 to 25) were sampled from each plot due to poor growing conditions that resulted in incomplete ear development that impeded accurate injury measurement.

#### Stalk Sampling

In 2023, sweet and grain corn plants were collected from all Bt and non-Bt sentinel plots at three sites (Winchester, ON, Ridgetown, ON, and Sussex, NB) in mid-late Oct at the R5 to R6 stage for evaluation of stalk injury by *O. nubilalis*. Stalk sampling was not conducted at sentinel sites in NB and QC due to labor limitations. Ten consecutive plants were selected from each of the central two rows of each plot and cut at the soil line using pruning shears. The number of borer holes on each plant was recorded, and stalks were dissected lengthwise from tassel to soil line. The length of each tunnel formed by *O. nubilalis* feeding was measured, and the number and instar of *O. nubilalis* larvae within each tunnel were recorded. The production of Bt toxins in plants that contained *O. nubilalis* larvae was confirmed at the time of sampling using leaf tissue and EnviroLogix QuickStix kits.

#### Bioassays

##### Insect strains and rearing

Ambient populations of *O. nubilalis* larvae were collected from sentinel plots of sweet and grain corn and were also collected from commercial fields of grain corn and other host crops such as potatoes (*Solanum tuberosum* L.), millet (*Panicum miliaceum* L.), and various vegetables in MB, ON, QC, NB, PEI, and NS (Figs 1 and 2) where sentinel plots were not planted to expand the scope of resistance monitoring. Crops were visually examined for evidence of the presence of *O. nubilalis*, stalks split to verify larvae were present, and portions of stalks and stems containing *O. nubilalis* were shipped to the University of Guelph Ridgetown Campus, Ridgetown, ON laboratory, and reared as described in Smith et al. (2019) and Smith and Farhan (2023). Meridic diet used for rearing and bioassays was prepared following the recipe described by Guthrie (1985). Collections were comprised of fifth-instar larvae except for the Brooklyn, NS strain, which was established from three egg masses producing ∼35 larvae. Susceptible strains originally collected from non-Bt corn near Delaware, ON, in 2016 (hereafter referred to as Delaware-Sus ON), Fletcher, ON, in 2020 (hereafter referred to as Fletcher-Sus ON), and from potatoes near Ste. Marguerite-Marie, QC, in 2019 (hereafter referred to as St. Marguerite-Marie-Sus QC) were maintained in the laboratory without exposure to Bt toxins and were used as controls (Fig. 1).

##### Diet overlay bioassays

Concentration response diet-overlay bioassays were conducted with Cry1Fa, Cry1Ab, Cry1A.105, and Cry2Ab toxins using established protocols (Marcon et al. 1999, Smith et al. 2019, Smith and Farhan 2023). Cry1Fa standards (>99% purity) were obtained from Corteva Agriscience (2021 to 2022) (Johnston, IA) and from Dr. Jurat-Fuentes (University of Tennessee, Knoxville, TN) in 2023; Cry1Ab, Cry1A.105, and Cry2Ab standards (91, 93, and 87% purity, respectively), were provided by Bayer CropScience (St. Louis, MO). Cry1Fa was serially diluted from the stock solution using 10 mM N-cyclohexyl-3-aminopropanesulfonic acid (CAPS) buffer, pH 10.5. Cry1Ab and Cry2Ab were serially diluted from their stock solutions using 0.1% Triton X-100 buffer. Cry1A.105 was diluted in 10 mM sodium carbonate/sodium bicarbonate buffer, pH 10.5. Non-Bt controls received the corresponding buffer.

Diet in 128-well trays (Frontier Agricultural Sciences, Newark, DE) was overlaid with 30 μL of the toxin solution per well (surface area 2.0 cm^2^). Tested concentrations were: Cry1Fa (0, 1.9, 3.8, 7.5, 15.0, 30.0, 60.0, and 200.0 ng cm^−2^); Cry1Ab (0, 0.4, 0.7, 1.4, 2.7, 5.6, 11.2, and 22.4 ng cm^−2^); Cry1A.105 (0, 0.5, 0.9, 1.9, 3.8, 7.5, 15.0, and 30.0 ng cm^−2^); and Cry2Ab (0, 5.6, 11.3, 22.5, 45.0, 90.0, and 180.0 ng Cry2Ab cm^−2^). Trays were manually tilted in all directions to distribute the toxin over the surface area of the diet air-dried. One neonate (<24 h old) was placed per well using a fine-point paint brush, and trays were sealed with ventilated adhesive lids (Frontier Agricultural Sciences, Newark, DE). Bioassays were maintained at 26 °C (light)/18 °C (dark), 60% RH, and 16:8 h L:D photoperiod; trays were covered to reduce light and encourage feeding. Mortality and individual larval weights were recorded after 7 d; larvae unresponsive to prodding or ≤0.1 mg and undeveloped beyond first instar were considered dead (Marcon et al. 1999, Smith et al. 2019, Smith and Farhan 2023). Each concentration included 24 to 32 larvae, and bioassays were replicated at least twice per strain and toxin, depending on larval availability.

##### Leaf tissue bioassays

Corn leaf tissue bioassays were conducted with *O. nubilalis* collected in 2022 and 2023 as described in Smith et al. (2019) to simulate *in planta* Bt exposure under controlled conditions. Both vegetative (V6-9) and reproductive (R1-R2) (Abendroth et al. 2011) stage tissues were tested because previous studies reported greater survival of Cry1-resistant larvae on reproductive tissue, likely due to reduced Bt production in mature plants (Pereira et al. 2008, Crespo et al. 2009). Additionally, because the voltinism of *O. nubilalis* in eastern Canada remains uncertain, larvae may encounter either vegetative or reproductive stages in the field, making it relevant to evaluate both tissue types in bioassays.

In 2022, vegetative-stage bioassays were performed on Cry1Ab, Cry1A.105, Cry2Ab, and non-Bt cultivars using strains from St. Armand (*F*_8_), St. Mathieu-de-Beloeil, QC (*F*_8_), and Carman, MB (*F*_12, 13_). Reproductive-stage assays included St. Armand (*F*_8–12_) and St. Georges, PEI (*F*_11_). Additional vegetative and reproductive assays were conducted on Cry1Fa, Cry1Fa + Cry1Ab, and non-Bt cultivars with strains from St. Armand (*F*_6, 7, 12_), St. Mathieu-de-Beloeil (*F*_7_), St. Georges (*F*_9, 10_), Truro, NS (*F*_8–10_), Sussex, NB (*F*_9, 10, 15_), and Carman (*F*_7, 8, 15_). In 2023, Clifton, NS (*F*_11, 14_) and Salisbury A, NB (*F*_9_) strains were tested on Bayer cultivars; Salisbury A (*F*_9_) and Sussex (*F*_8–10_) were tested on Cry1Fa treatments, while Clifton (*F*_11_) was tested only on reproductive tissue due to limited larvae. The Delaware-Sus ON laboratory strain served as the susceptible control. Each bioassay was replicated 2 or 3 times, depending on larval availability.

Leaves were collected from greenhouse-grown plants in 8 L pots, washed with 95% ethanol, rinsed in distilled water, and cut into 4 cm^2^ pieces containing the midrib. Leaf pieces were placed in 32-well trays (Frontier Agricultural Sciences, Newark, DE) lined with 2.5 mL of 5% agar and filter paper. Each replicate included 16 wells per treatment (non-Bt (Hybrid A), Cry1Fa (Hybrid B), Cry1Fa + Cry1Ab (Hybrid C)) cultivars provided by Pioneer Hi-Bred Production Co. or non-Bt, Cry1Ab, Cry1A.105, or Cry2Ab cultivars from non-commercial seed lots provided by Bayer CropScience). Five unfed neonates were placed on each leaf piece using a sterilized fine-point brush, and trays were sealed with ventilated adhesive lids (Frontier Agricultural Sciences, Newark, DE ). Mortality was assessed at 7 days after introduction (DAI); larvae unresponsive to gentle prodding were considered dead. Surviving larvae in each well were weighed as a cohort at 7 DAI.

### Data Analysis

#### Evaluation of *Ostrinia nubilalis* Injury at Sentinel Sites

The results of ear assessments are provided as descriptive statistics (proportion of plants with ear injury and number of larvae observed) in Supplementary Table S2. For stalk sampling, means and standard errors were calculated for each site and cultivar for the proportion of plants with stalk injury, total tunnel length per plant, number of borer holes per plant, and number of larvae per plant.

#### Diet Overlay Bioassays

Mortality data were analyzed using PROC PROBIT in SAS 9.4 (SAS Institute Inc., Cary, NC) to estimate the slope of the concentration-response curve, LC_50_ value (the concentration of toxin causing mortality of 50% of the individuals in a strain), and 95% confidence intervals. The OPTC option was used to estimate mortality due to natural variation in each strain; replicates with ≥25% mortality in the control were excluded from analysis. LC_50_ values were considered significantly different if their confidence intervals did not overlap.

Resistance ratios were calculated as the quotient of the LC_50_ values of each field-derived strain and susceptible laboratory strain (Tabashnik et al. 2014). If an LC_50_ value could not be estimated for a strain, the greatest concentration tested was used as the numerator. Proportional survival at the greatest concentration of each toxin was calculated using Abbott’s correction (Abbott 1925) and compared among field-derived and laboratory stains, within each year using PROC GLIMMIX. Strain and replication were considered fixed and random effects, respectively, and LSMeans were compared using Tukey’s HSD test. Proportional survival data followed a normal distribution.

#### Leaf Tissue Bioassays

Proportional survival was calculated as the number of surviving larvae at 7 DAI divided by the number of larvae initially introduced to each well. Mean larval weight at 7 DAI was calculated by dividing the total weight of larvae within a well by the number of surviving larvae. The effect of Bt toxin, field-derived strain, and their interaction on proportional survival was analyzed using PROC GLIMMIX with well within replicate as a random effect. Survival was analyzed separately for Corteva AgriScience and Bayer CropScience cultivars and vegetative and reproductive tissue. Proportional survival data was transformed using arcsine square-root (*x*) and analyzed using a normal distribution, and larval weight data followed a normal distribution. LSMeans were compared using Tukey’s HSD test (α *<* 0.05), and backtransformed means and standard errors are presented for proportional survival. The proportion of first, second, and third instar surviving larvae observed at 7 DAI is reported in Supplemental Table S3.

## Results

### Ear Sampling

From 2019 to 2023, *O. nubilalis* ear injury and larval presence were generally low across sentinel sites in NS, NB, QC, and ON. At Berwick, NS, in 2020, 16% of Cry1Fa grain corn ears exhibited 1 to 3 cm^2^ kernel injury and six larvae were observed. At Cambridge, NS, in 2022, minor injury occurred on non-Bt sweet corn (Obsession I), and non-Bt and Cry1Fa grain corn (≤2% of ears), with only one larva observed on non-Bt sweet corn (Supplementary Table S2).

At St. Mathieu-de-Beloeil, QC, no ear injury was observed in 2020, 2021, or 2023. In 2022, 22% of non-Bt ears (0.3 to 13.0 cm^2^ injury) and 12% of Cry1Ab ears (1.3 to 2.0 cm^2^) were detected with one larva in each ear (Supplementary Table S2). 32% of Cry1Fa ears had 3.1 cm^2^ of kernel injury, and 12 larvae were observed (Supplementary Table S2).

At Winchester, ON, Bt sweet corn was largely uninjured except for minimal feeding by one larva on a Cry1Ab ear in 2021. In 2023, injury was observed in the grain corn hybrids. From the first planting date, three Cry1Fa and 7 Cry1Fa + Cry1Ab plants sustained 1.0 to 15.0 and 0.1 to 3.0 cm^2^ injury, respectively. From the second planting date, one Cry1Fa (7 cm^2^) and four Cry1Fa + Cry1Ab plants (range 1 to 8 cm^2^), were injured. *O. nubilalis* larvae were only recovered from non-Bt ears (Supplementary Table S2).

At Sussex, NB, ear injury was absent in 2021 except for one larva observed on Cry1Ab sweet corn (Supplementary Table S2). In 2022, minor injury and one larva were observed on Cry1Ab sweet corn and on Cry1Fa grain corn (Supplementary Table S2). In 2023, severe injury by *O. nubilalis* in ‘Providence’ non-Bt sweet corn resulted in poor ear production and lodging and prevented ear assessment; injury and larvae were observed on ‘Obsession I’ non-Bt sweet corn and non-Bt grain corn (Supplementary Table S2).

At Ridgetown, ON, ear injury was absent from 2019 to 2023 except for minimal feeding on a non-Bt ear in 2020 (Supplementary Table S2). No injury or larvae were observed at Freetown, PEI, in 2021 (Supplementary Table S2). No feeding or *O. nubilalis* larvae were observed on ears of Cry1A.105 + Cry2Ab sweet corn at any site from 2019 to 2023 (Supplementary Table S2).

### Stalk Sampling

**Fig. 3.**
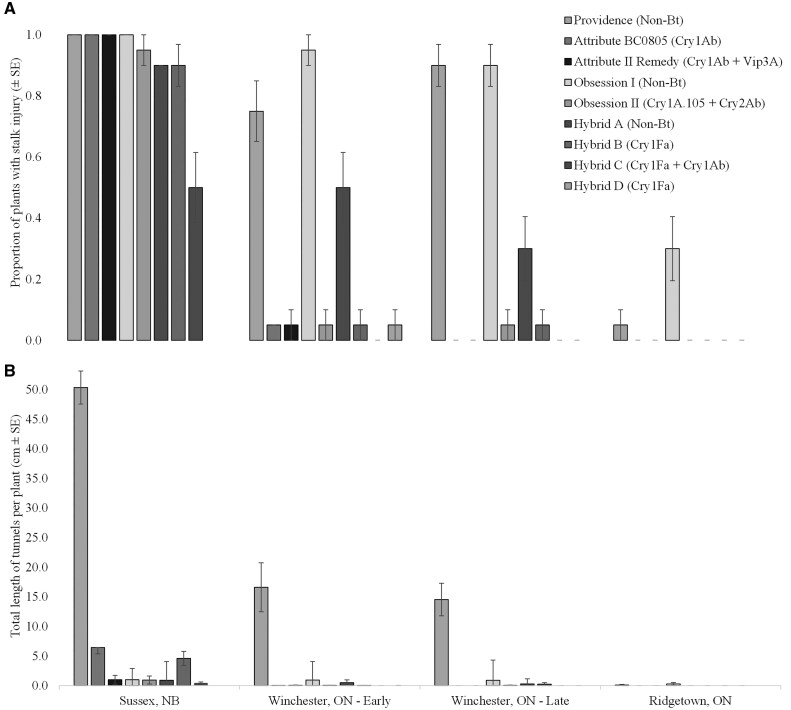
A) Incidence of stalk injury by *Ostrinia nubilalis* and B) total tunnel length per plant in Bt and non-Bt sweet and grain corn cultivars at sentinel sites in Canada in 2023.

**Fig. 4.**
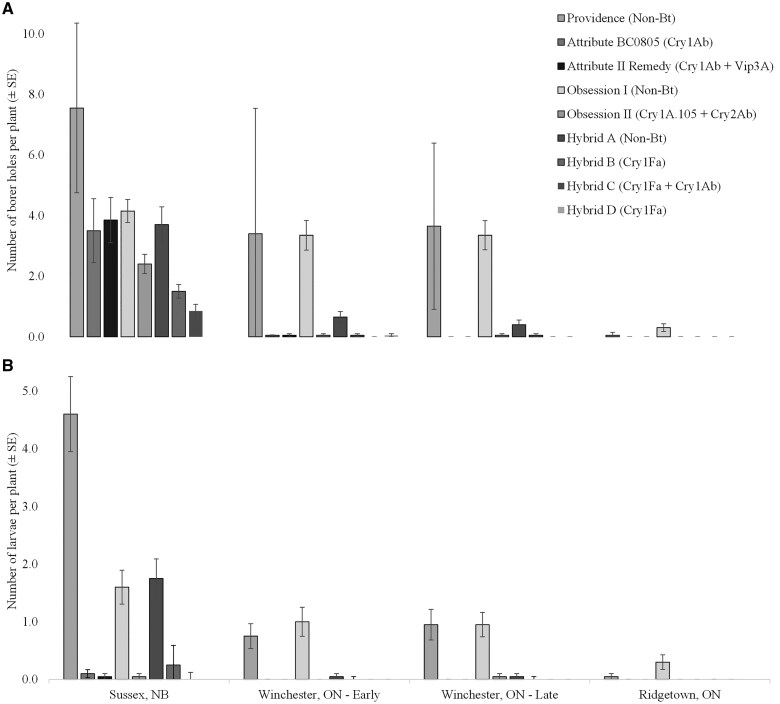
Number of A) borer holes per plant and B) *Ostrinia nubilalis* larvae per plant in Bt and non-Bt sweet and grain corn cultivars at sentinel sites in Canada in 2023.

In 2023, the greatest stalk injury occurred at the Sussex, NB sentinel site. All evaluated sweet corn plants of non-Bt, Cry1Ab, and Cry1Ab + Vip3A cultivars, and 95% of Cry1A.105 + Cry2Ab plants exhibited injury from *O. nubilalis* (Fig. 3A). Among grain corn, 90% of non-Bt and Cry1Fa and 50% of Cry1Fa + Cry1Ab plants had signs of stalk injury (Fig. 3A). The non-Bt sweet corn cultivar ‘Providence’ sustained the greatest injury, averaging 50.4 cm of tunneling, 7.5 borer holes, and 4.6 larvae per plant (Figs 3B and 4A and B). Injury in non-Bt sweet corn ‘Obsession I’ and grain corn ‘Hybrid A’ was approximately half as severe (Figs 3 and 4). Bt cultivars exhibited substantially less injury, though most were affected. Cry1Ab and Cry1Ab + Vip3A sweet corn cultivars sustained similar injury (6.5 cm of tunneling, 3.7 holes per plant, and 0.1 larvae per plant), slightly exceeding Cry1A.105 + Cry2Ab (4.2 cm tunnel length, 2.4 holes per plant, 0.05 larvae per plant). The Cry1Fa grain corn sustained, on average, 4.6 cm of tunneling, 1.5 holes, and 0.25 larvae per plant, while Cry1Fa + Cry1Ab plants had minimal injury with 0.5 cm of tunneling and 0.9 holes per plant on average and no larvae (Figs 3 and 4).

At the Winchester, ON site, *O. nubilalis* injury was detected on approximately 88% of non-Bt sweet corn plants, with similar injury levels across planting dates (Figs 3 and 4). Non-Bt sweet corn averaged 3.4 holes, 15.5 cm of tunneling, and 0.9 larvae per plant (Figs 3B and 4A and B). In early-planted sweet corn, one plant in 20 from each of the ‘Attribute’ (Cry1Ab) and ‘Remedy’ (Cry1Ab + Vip3A) plots exhibited a single borer hole and minimal tunneling (0.25 and 1.0 cm, respectively); no larvae were found, and no injury was observed in the late-planted plots (Figs 3 and 4). One Cry1A.105 + Cry2Ab plant from each planting date had one hole; one fifth-instar larva was found in a late-planted plant within a 1.0 cm tunnel (Figs 3 and 4). Among early-planted grain corn hybrids, 50% of non-Bt plants exhibited injury, averaging 0.7 borer holes, 1.7 cm of tunneling per plant; two fifth-instar larvae were recovered from 20 plants, whereas Cry1Fa hybrids each had one plant with a single hole and 0.25 cm of tunneling (Figs 3 and 4). In late-planted non-Bt grain corn, 30% of plants were injured, averaging 1.3 holes and 6.3 cm of tunneling per plant; one Cry1Fa (Hybrid B) plant had 5 cm of tunneling. No injury or larvae were detected in Cry1Fa + Cry1Ab plants at either planting date (Figs 3 and 4). At the Ridgetown, ON site, *O. nubilalis* injury was minimal and restricted to non-Bt cultivars (Figs 3 and 4).

### Diet Overlay Bioassays

Six of the 17 field-derived strains collected in 2021 to 2023 were resistant to Cry1Fa and Cry1A.105; two from each of the provinces of NS (Truro and Clifton), NB (Salisbury A and Sussex), and QC (St. Armand and St.-Mathieu-de-Beloeil, QC) (Tables 1 to 3). The strain collected from Truro, NS was also resistant to Cry1Ab with 55% survival at 22.4 ng Cry1Ab cm^−2^ and a RR > 20 (Table 2). The strains collected from Clifton, NS and Sussex, NB in 2023 also showed early warning of resistance to Cry1Ab with significantly greater LC_50_ values relative to the susceptible laboratory strain and RRs of 4.6 and 2.0, respectively, yet no survival was observed at the greatest concentration of Cry1Ab (Table 2).

**Table 1.** Response of Canadian field-derived strains of *Ostrinia nubilalis* collected in 2021 to 2023 to Cry1Fa insecticidal toxin in diet-overlay bioassays

Strain	n^a^	Slope ± SE	LC_50_ (95% CI)^b^	Resistance ratio^c^			Survival (% ±SE)^d^
2021				C1	C2	C3	
**Delaware-Sus, ON (*F*_46_)**	576	2.9 ± 0.30	6.53 (5.33 to 7.75)a^e^	1.0	1.2	.	0
**Fletcher-Sus, ON (*F*_9_)**	576	3.0 ± 0.39	5.40 (4.22 to 6.48)a	0.8	1.0	.	0
**Merlin, ON (*F*_4_)**	571	2.1 ± 0.30	6.33 (4.11 to 8.49)a	1.0	1.2	.	0
**Carman, MB (*F*_3_)**	576	4.3 ± 1.57	13.10 (3.77 to 16.97)a	2.0	2.4	.	0
**Wellwood, MB (*F*_2_)**	576	2.4 ± 0.30	6.54 (4.83 to 8.19)a	1.0	1.2	.	0
***P*-value**							1.0000
**2022**							
**Delaware-Sus, ON (*F*_54_)**	768	2.6 ± 0.26	5.86 (4.71 to 7.01)a	1.0	1.0	.	0.0 ± 2.32a
**Fletcher-Sus, ON (*F*_18_)**	754	2.2 ± 0.21	5.79 (4.59 to 7.00)a	1.0	1.0	.	0.0 ± 2.32a
**St. Georges, PEI (*F*_4_)**	714	3.1 ± 0.54	10.92 (7.64 to 13.45)b	1.9	1.9	.	0.0 ± 2.32a
**Granville, PEI**	.^f^	.	.	.	.	.	.
**Port Williams, NS (*F*_9_)**	761	21.7 ± 0.41	5.78 (3.37 to 8.50)ab	1.0	1.0	.	1.1 ± 2.32a
**Truro, NS (*F*_3_)**	721	NA	>200	>34.1	>34.5	.	84.7 ± 2.32b
**Canning, NS (*F*_3_)**	764	2.7 ± 0.29	6.24 (4.97 to 7.45)ab	1.1	1.1	.	0.0 ± 2.32a
**Ward’s Creek, NB (*F*_4, 9_)**	733	1.9 ± 0.23	7.62 (5.20 to 10.02)ab	1.3	1.3	.	0.0 ± 2.32a
**Sussex, NB (*F*_4_)**	761	1.0 ± 0.10	6.47 (4.40 to 8.90)ab	1.1	1.1	.	11.5 ± 2.32a
**St. Armand, QC (*F*_5_)**	756	NA	>200	>34.1	>34.5	.	91.5 ± 2.32bc
**Saint-Mathieu-de-Beloeil, QC (*F*_1_)**	742	NA	>200	>34.1	>34.5	.	100.0 ± 2.32c
**Merlin, ON (*F*_6_)**	768	2.8 ± 0.38	6.56 (4.76 to 8.13)ab	1.1	1.1	.	0.0 ± 2.32a
**Winchester, ON**	.^f^	.	.	.	.	.	.
**Carman, MB (*F*_6-7_)**	722	1.8 ± 0.43	13.03 (4.47 to 20.33)ab	2.2	2.3	.	0.0 ± 2.32a
***P*-value**							<0.0001
**2023**							
**Delaware-Sus, ON (*F*_64_)**	728	2.8 ± 0.75	1.19 (0.38 to 1.76)a	1.0	.	1.3	0.0 ± 6.83a
**Ste. Marguerite-Marie-Sus, QC (*F*_28_)**	724	3.4 ± 0.98	0.94 (0.28 to 1.34)a	0.8		1.0	0.0 ± 6.83a
**Clifton, NS (*F*_11_)**	766	0.09 ± 0.31	>200	>168.1	.	>212.8	97.9 ± 6.83 b
**Salisbury, NB (A) (*F*_8,10_)**	761	0.24 ± 0.31	>200	>168.1	.	>212.8	93.3 ± 6.83 b
**Salisbury, NB (B) (*F*_5_)**	745	3.5 ± 0.62	1.58 (1.13 to 1.91)a	0.8	.	1.7	0.0 ± 6.83a
**Sussex, NB (*F*_5,7_)**	504	0.19 ± 0.26	>200	>168.1	.	>212.8	62.6 ± 8.37 b
***P*-value**							<0.0001

a *n* is the total number of larvae infested in bioassay; 24 to 32 larvae were exposed to each concentration, and bioassays were replicated at least three times per strain.

b Lethal concentration of Cry1F (ng/cm^2^) estimated to cause 50% mortality within strain (95% confidence intervals).

c Resistance ratio = LC_50_ value of the field-derived strain divided by the LC_50_ value of the Cry1Ab-susceptible laboratory strain. C1 = Delaware-Sus, ON, C2 = Fletcher-Sus, ON, C3 = Ste. Marguerite-Marie-Sus, QC.

d Corrected survival at 200.0 ng Cry1F per cm^2^ diet. LSMeans followed by the same letter are not significantly different according to Tukey’s HSD test (*P *> 0.05).

e Values followed by the same letter within years are not significantly different based on overlapping 95% confidence intervals.

f Collection did not produce enough offspring to complete evaluation.

**Table 2.** Response of Canadian field-derived strains of *Ostrinia nubilalis* collected in 2021 to 2023 to Cry1Ab insecticidal toxin in diet-overlay bioassays

Strain	*n* ^a^	Slope ± SE	LC_50_ (95% CI)^b^	Resistance ratio^c^			Survival (% ± SE)^d^
**2021**				C1	C2	C3	
**Delaware-Sus, ON (*F*_37_)**	430	3.7 ± 0.92	0.95 (0.51 to 1.22)a^e^	1.0	0.9	.	0.0 ± 0.62
**Fletcher-Sus, ON (*F*_2_)**	429	2.9 ± 0.85	1.11 (0.31 to 1.66)ab	1.2	1.0	.	0.0 ± 0.62
**Merlin, ON (*F*_1_)**	432	2.4 ± 0.31	2.06 (1.52 to 2.56)b	2.2	1.9	.	0.0 ± 0.62
**Carman, MB (*F*_1_)**	429	2.5 ± 0.33	2.22 (1.62 to 2.79)b	2.3	2.0	.	0.0 ± 0.62
**Wellwood, MB (*F*_1-2_)**	432	2.8 ± 0.39	2.10 (1.55-2.62)b	2.2	1.9	.	0.0 ± 0.62
***P*-value**							0.4609
**2022**							
**Delaware-Sus, ON (*F*_52_)**	711	3.2 ± 1.02	0.56 (0.22-0.90)ab	1.0	0.5	.	0.0 ± 5.87a
**Fletcher-Sus, ON (*F*_14_)**	680	2.1 ± 0.31	1.09 (0.69-1.52)bcd	1.9	1.0	.	0.0 ± 5.87a
**St. Georges, PEI (*F*_2_)**	729	3.5 ± 0.39	0.82 (0.68-0.96)bc	1.4	0.8	.	0.0 ± 5.87a
**Granville, PEI (*F*_2_)**	703	3.2 ± 1.41	0.80 (0.00-1.84)a-d	1.4	0.7	.	0.0 ± 5.87a
**Port Williams, NS (*F*_6_)**	665	2.1 ± 0.49	0.30 (0.11-0.47)a	0.5	0.3	.	0.0 ± 5.87a
**Truro, NS (*F*_2_)**	981	1.1 ± 0.61	>22.4	>40	>20.6	.	55.0 ± 5.87b
**Canning, NS (*F*_3_)**	700	1.9 ± 0.62	0.95 (0.14-2.28)a-d	1.7	0.9	.	7.5 ± 5.87a
**Ward’s Creek, NB (*F*_1_)**	667	2.0 ± 0.47	1.10 (0.41-1.91)a-d	2.0	1.0	.	0.0 ± 5.87a
**Sussex, NB (*F*_2_)**	719	3.6 ± 0.79	1.45 (0.94-1.81)cd	2.6	1.3	.	0.0 ± 5.87a
**St. Armand, QC (*F*_1_)**	768	2.2 ± 0.19	1.41 (1.13-1.71)d	2.5	1.3	.	7.1 ± 5.87a
**Saint-Mathieu-de-Beloeil, QC (*F*_1, 5_)**	747	1.7 ± 0.31	2.41 (1.30-3.75)d	4.3	2.2	.	6.7 ± 5.87a
**Merlin, ON (*F*_3_)**	720	3.1 ± 0.35	0.72 (0.58-0.86)b	1.3	0.7	.	0.0 ± 5.87a
**Winchester, ON**	.^f^	.	.	.	.	.	.
**Carman, MB (*F*_1, 6, 7_)**	724	2.1 ± 0.47	0.59 (0.24-0.94)abc	1.1	0.5	.	0.0 ± 5.87a
***P*-value**							<0.0001
**2023**							
**Ste. Marguerite-Marie-Sus, QC (*F*_28_)**	730	3.4 ± 0.48	0.35 (0.28-0.42)a	.	.	1.0	0.0 ± 0.00
**Clifton, NS (*F*_7, 10_)**	768	4.9 ± 0.71	1.62 (1.37-1.82)c	.	.	4.6	0.0 ± 0.00
**Salisbury, NB (A) (*F*_8, 10_)**	739	1.8 ± 0.57	0.59 (0.07-1.22)ab	.	.	1.7	0.0 ± 0.00
**Salisbury, NB (B) (*F*_3, 6_)**	737	1.9 ± 0.85	0.21 (0.00-0.29)a	.	.	0.6	0.0 ± 0.00
**Sussex, NB (*F*_2,7_)**	979	2.1 ± 0.19	0.69 (0.55-0.83)b	.	.	2.0	0.0 ± 0.00
***P*-value**							.

a *n* = total number of larvae infested in bioassay; 24 to 32 larvae were exposed to each concentration and bioassays were replicated at least three times per strain.

b Lethal concentration of Cry1Ab (ng/cm^2^) estimated to cause 50% mortality within strain (95% confidence intervals).

c Resistance ratio = LC_50_ value of the field-derived strain divided by the LC_50_ value of the Cry1Ab-susceptible laboratory strain. C1= Delaware-Sus, ON, C2= Fletcher-Sus, ON, C3= Ste. Marguerite-Marie-Sus, QC.

d Corrected survival at 22.4 ng Cry1Ab per cm^2^ diet. LSMeans followed by the same letter are not significantly different according to Tukey’s HSD test (*P *> 0.05).

e Values followed by the same letter within years are not significantly different based on overlapping 95% confidence intervals.

f Collection did not produce enough offspring to complete evaluation.

**Table 3.** Response of Canadian field-derived strains of *Ostrinia nubilalis* collected in 2021 to 2023 to Cry1A.105 insecticidal toxin in diet-overlay bioassays

Strain	*n* ^a^	Slope ± SE	LC_50_ (95% CI)^b^	Resistance ratio^c^		Survival (% ± SE)^d^
**2021**				C1	C2	
**Delaware-Sus, ON (*F*_38_)**	430	2.4 ± 2.44	2.79 (1.30 to 4.21)a^e^	1.0	0.8	0.0 ± 2.17
**Fletcher-Sus, ON (*F*_2_)**	430	3.3 ± 0.61	3.37 (2.28 to 4.19)a	1.2	1.0	0.0 ± 2.17
**Merlin, ON (*F*_4_)**	424	3.2 ± 0.37	9.42 (7.68 to 11.13)b	3.4	2.8	6.0 ± 2.17
**Carman, MB (*F*_5_)**	415	2.6 ± 0.33	7.87 (5.90 to 9.81)b	2.3	2.3	7.1 ± 2.17
**Wellwood, MB (*F*_5_)**	412	3.0 ± 0.40	7.79 (6.03 to 9.45)b	2.3	2.3	0.0 ± 2.17
***P*-value**						0.1032
**2022**						
**Delaware-Sus, ON (*F*_52_)**	765	2.5 ± 0.30	1.03 (0.79 to 1.28)a	1.0	0.7	0.0 ± 5.69a
**Fletcher-Sus, ON (*F*_14_)**	767	2.8 ± 0.30	1.46 (1.13 to 1.81)ab	1.4	1.0	0.0 ± 5.69a
**St. Georges, PEI (*F*_2_)**	761	2.7 ± 0.22	1.50 (1.29 to 1.73)b	1.5	1.0	0.0 ± 5.69a
**Granville, PEI (*F*_2_)**	761	4.4 ± 0.91	1.96 (1.41 to 2.31)b	1.0	1.3	0.0 ± 5.69a
**Port Williams, NS (*F*_6_)**	762	2.1 ± 0.31	2.13 (1.36 to 2.92)bc	2.1	1.5	2.2 ± 5.69a
**Truro, NS (*F*_2_)**	1011	1.1 ± 0.36	>30.0	>29.1	>20.5	69.4 ± 4.92b
**Canning, NS (*F*_3_)**	766	2.1 ± 0.26	1.23 (0.87 to 1.61)ab	1.2	0.8	1.0 ± 5.69a
**Ward’s Creek, NB (*F*_1_)**	765	4.1 ± 1.01	1.80 (1.09 to 2.27)ab	1.7	1.2	0.0 ± 5.69a
**Sussex, NB (*F*_2_)**	766	1.9 ± 0.27	1.22 (0.75 to 1.74)ab	1.2	0.8	0.0 ± 5.69a
**St. Armand, QC (*F*_5_)**	757	0.42 ± 0.30	>30.0	>29.1	>20.5	69.1 ± 5.69 b
**Saint-Mathieu-de-Beloeil, QC (*F*_5_)**	1000	0.46 ± 0.27	>30.0	>29.1	>20.5	75.9 ± 4.92 b
**Merlin, ON (*F*_3_)**	759	4.2 ± 0.49	2.75 (2.40 to 3.08)c	2.7	1.9	0.0 ± 5.69a
**Winchester, ON**	.^f^	.	.	.	.	.
**Carman, MB (*F*_1-2_)**	768	2.1 ± 0.18	1.05 (0.87 to 1.24)a	1.0	0.7	0.0 ± 5.69a
***P*-value**						<0.0001
**2023**						
**Delaware-Sus, ON (*F*_63_)**	730	4.5 ± 1.55	0.25 (0.09 to 0.33)a	1.0	.	2.6 ± 10.66
**Clifton, NS (*F*_10, 11_)**	764	2.9 ± 0.42	6.67 (5.06 to 8.19)b	26.7	.	16.1 ± 10.66
**Salisbury, NB (A) (*F*_3, 8_)**	748	0.4 ± 0.48	>18.0	>72.0	.	33.1 ± 10.33
**Salisbury, NB (B) (*F*_3, 5_)**	717	1.5 ± 0.43	0.41 (0.06 to 0.88)a	1.6	.	6.3 ± 10.66
**Sussex, NB (*F*_6,7_)**	762	0.8 ± 0.30	3.47 (0.24 to 21.29)ab	13.9	.	20.3 ± 10.66
***P*-value**						0.2703

a *n* = total number of larvae infested in bioassay; 24 to 32 larvae were exposed to each concentration and bioassays were replicated at least three times per strain.

b Lethal concentration of Cry1A.105 (ng/cm^2^) estimated to cause 50% mortality within strain (95% confidence intervals).

c Resistance ratio = LC_50_ value of the field-derived strain divided by the LC_50_ value of the Cry1Ab-susceptible laboratory strain. C1 = Delaware-Sus, ON, C2 = Fletcher-Sus, ON.

d Corrected survival at 30.0 ng Cry1A.105 per cm^2^ diet in 2021 to 2022 and 18.0 ng Cry1A.105 per cm^2^ diet in 2023 except for Clifton, NS for which the greatest concentration tested was 15.0 ng Cry1A.105 per cm^2^ diet. LSMeans followed by the same letter are not significantly different according to Tukey’s HSD test (*P *> 0.05).

e Values followed by the same letter within years are not significantly different based on overlapping 95% confidence intervals.

f Collection did not produce enough offspring to complete evaluation.

The field-derived strains collected in 2022 from St. Armand and St.-Mathieu-de-Beloeil, QC, were resistant to Cry1Fa and Cry1A.105 with RRs greater than 34 and 20 for the respective toxins (Tables 1 and 3). A strain collected from St. Georges, PEI, in 2022 had a significantly greater LC_50_ value for Cry1Fa compared to the susceptible laboratory strains and a RR of 1.9 (Table 1). For Cry1A.105, both field-derived strains collected in MB in 2021, and the strains collected from Merlin, ON, in 2021 and 2022 had significantly greater LC_50_ values relative to the susceptible laboratory strains with RRs ranging from 1.9 to 3.4 (Table 3). None of the field-derived strains tested for Cry2Ab susceptibility showed evidence of resistance; however, the 2022 St.-Mathieu-de-Beloeil, QC population had elevated survival and RR values of 1.7 and 2.2 (Table 4).

**Table 4.** Response of Canadian field-derived strains of *Ostrinia nubilalis* collected in 2021 to 2023 to Cry2Ab insecticidal toxin in diet-overlay bioassays

Strain	*n* ^a^	Slope ± SE	LC_50_ (95% CI)^b^	Resistance ratio^c^			Survival (% ± SE)^d^
**2021**				C1	C2	C3	
**Delaware-Sus, ON (*F*_37_)**	432	3.2 ± 0.47	14.36 (11.11 to 17.27)ab^e^	1.0	1.7	.	0.0 ± 0.00
**Fletcher-Sus, ON (*F*_2_)**	432	2.2 ± 0.38	8.22 (4.73 to 11.24)a	0.6	1.0	.	0.0 ± 0.00
**Merlin, ON (*F*_4_)**	431	3.0 ± 0.38	16.08 (12.91 to 19.05)b	1.1	2.0	.	0.0 ± 0.00
**Carman, MB (*F*_4_)**	427	2.6 ± 0.32	16.87 (13.28 to 20.32)b	1.2	2.1	.	0.0 ± 0.00
**Wellwood, MB (*F*_4_)**	428	2.9 ± 0.35	16.60 (13.64 to 19.45)b	1.2	2.0	.	0.0 ± 0.00
***P*-value**							1.0000
**2022**							
**Delaware-Sus, ON (*F*_53_)**	636	2.4 ± 0.28	15.71 (11.89 to 19.59)a	1.0	0.8	.	0.0 ± 2.20a
**Fletcher-Sus, ON (*F*_18_)**	639	2.4 ± 0.32	19.63 (14.02 to 25.20)ab	1.2	1.0	.	0.0 ± 2.20a
**St. Georges, PEI (*F*_3_)**	666	2.8 ± 0.38	15.87 (11.54 to 19.89)a	1.0	0.8	.	0.0 ± 2.20a
**Granville, PEI (*F*_2_)**	664	2.7 ± 0.29	17.19 (13.60 to 20.72)a	1.1	0.9	.	0.0 ± 2.20a
**Port Williams, NS (*F*_9_)**	667	1.7 ± 0.23	17.63 (11.64 to 24.21)ab	1.1	0.9	.	3.2 ± 2.20a
**Truro, NS (*F*_3_)**	896	2.7 ± 0.32	14.29 (10.79 to 17.83)a	0.9	0.7	.	0.8 ± 2.20a
**Canning, NS (*F*_6_)**	668	2.2 ± 0.22	17.54 (13.76 to 21.32)ab	1.1	0.9	.	0.0 ± 2.20a
**Ward’s Creek, NB (*F*_3_)**	668	2.9 ± 0.40	17.54 (13.23 to 22.33)ab	1.1	0.9	.	0.0 ± 2.20a
**Sussex, NB (*F*_9-10_)**	621	1.7 ± 0.31	2.68 (1.16 to 4.18)a	0.2	0.1	.	0.0 ± 2.20a
**St. Armand, QC (*F*_3-4_)**	636	1.7 ± 0.18	14.96 (10.80 to 19.34)a	1.0	0.8	.	4.6 ± 2.20a
**Saint-Mathieu-de-Beloeil, QC (*F*_1, 5_)**	661	1.1 ± 0.26	33.99 (13.46 to 67.33)ab	2.2	1.7	.	18.0 ± 2.20b
**Merlin, ON (*F*_4_)**	666	2.5 ± 0.45	33.25 (21.23 to 47.70)b	2.1	1.7	.	0.0 ± 2.20a
**Winchester, ON**	.^f^	.	.	.	.	.	.
**Carman, MB (*F*_1_)**	651	2.2 ± 0.19	18.88 (15.53 to 22.35)ab	1.2	1.0	.	0.0 ± 2.20a
***P*-value**							0.0002
**2023**							
**Ste. Marguerite-Marie-Sus, QC (*F*_31_)**	663	3.1 ± 0.50	7.68 (5.57 to 9.61)b	.	.	1.0	0.0 ± 0.00
**Clifton, NS (*F*_6, 11_)**	667	2.8 ± 0.32	18.51 (14.60 to 22.25)c	.	.	2.4	0.0 ± 0.00
**Salisbury, NB (A) (*F*_3, 8_)**	639	1.9 ± 0.33	11.79 (6.71 to 17.19)bc	.	.	1.5	0.0 ± 0.00
**Salisbury, NB (B) (*F*_3_)**	621	1.7 ± 0.30	6.74 (3.07 to 10.54)ab	.	.	0.9	0.0 ± 0.00
**Sussex, NB (*F*_5_,_6_)**	659	2.3 ± 0.30	5.81 (4.03 to 7.45)ab	.	.	0.8	0.0 ± 0.00
***P*-value**							.

a *n* = total number of larvae infested in bioassay; 24 to 32 larvae were exposed to each concentration and bioassays were replicated at least three times per strain.

b Lethal concentration of Cry2Ab (ng/cm^2^) estimated to cause 50% mortality within the strain (95% confidence intervals).

c Resistance ratio = LC_50_ value of the field-derived strain divided by the LC_50_ value of the Cry1Ab-susceptible laboratory strain. C1 = Delaware-Sus, ON, C2 = Fletcher-Sus, ON, C3 = Ste. Marguerite-Marie-Sus, QC.

d Corrected survival at 180.0 ng Cry2Ab per cm^2^ diet. LSMeans followed by the same letter are not significantly different according to Tukey’s HSD test (*P *> 0.05).

e Values followed by the same letter within years are not significantly different based on overlapping 95% confidence intervals.

f Collection did not produce enough offspring to complete evaluation.

### Leaf Tissue Bioassays

**Fig. 5.**
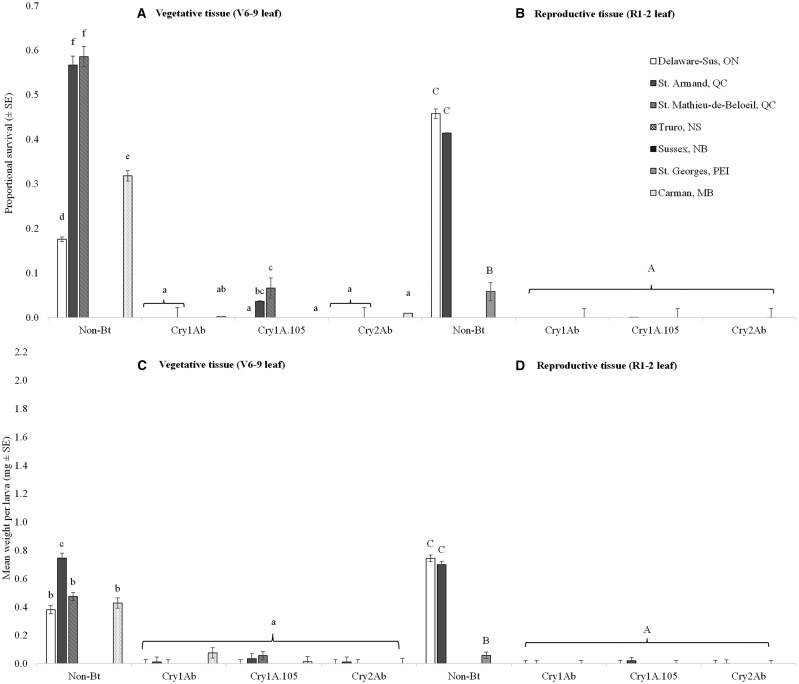
Proportional survival of *Ostrinia nubilalis* larvae from field-derived strains in Canada in 2022 after 7 d of exposure to A) vegetative or B) reproductive-stage leaf tissue and mean weight of surviving larvae on C) vegetative or D) reproductive-stage leaf tissue from non-Bt, Cry1Ab, Cry1A.105, or Cry2Ab corn. LSMEANS followed by the same letter within plant stage were not significantly different according to Tukey’s HSD test (*P *< 0.05).

For the 2022 field-derived strains, proportional survival and mean weight of *O. nubilalis* on Cry1Ab, Cry1A.105, Cry2Ab at 7 DAI depended on strain due to significant interactions for vegetative (survival: F_9,563_=12.41, *P *< 0.0001; weight: *F*_9,562_ = .40, *P *< 0.0001) and reproductive tissues (survival: *F*_6,502_ = 34.96, *P *< 0.0001; weight: *F*_6, 532_ = 9.35, *P *< 0.0001) (Fig. 5A to D). Survival of St. Armand and St. Mathieu-de-Beloeil, QC on Cry1A.105 vegetative tissue was approximately 3 to 6%, and significantly below non-Bt (Fig. 5A). Survivors on Cry1Ab or Cry1A.105 were predominantly first instars (apart from one second instar from St. Armand, QC) and weighed significantly less than those on non-Bt tissue (Fig. 5A and B). No survival was observed on reproductive stage Bt tissue apart from two larvae; one first and one second instar from St. Armand, QC on Cry1A.105 (Fig. 5B, Supplementary Table S3).

**Fig. 6.**
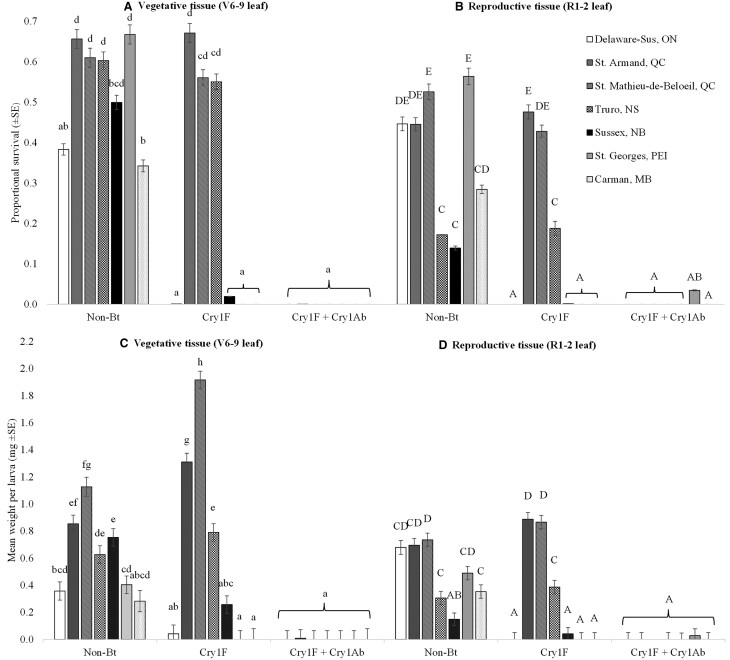
Proportional survival of *Ostrinia nubilalis* larvae from field-derived strains in Canada in 2022 after 7 d of exposure to A) vegetative or B) reproductive-stage leaf tissue and mean weight of surviving larvae C) vegetative or D) reproductive-stage leaf tissue from non-Bt, Cry1Fa, or Cry1Fa + Cry1Ab corn. LSMEANS followed by the same letter within plant stage were not significantly different according to Tukey’s HSD test (*P *< 0.05).

Proportional survival and mean weight of *O. nubilalis* on Cry1Fa, Cry1Fa + Cry1Ab, or non-Bt varied by strain for vegetative (survival: *F*_12, 880_ = 47.27, *P *< 0.0001; weight: *F*_12, 880_ = 36.47, *P *< 0.0001) and reproductive tissue (survival: *F*_10, 935_ = 44.81 *P *< 0.0001; weight: *F*_10, 795_ = 24.55, *P *< 0.0001) (Fig. 6A to D). Survival on Cry1Fa did not differ from non-Bt for St. Armand and St. Mathieu-de-Beloeil, QC and Truro, NS regardless of plant stage (Fig. 6A and B). The Sussex, NB strain showed approximately 0.2% survival on Cry1Fa vegetative tissue, significantly below non-Bt (Fig. 6A and B). No survival occurred on Cry1Fa + Cry1Ab except for St. Georges, PEI (∼3% on reproductive tissue) (Fig. 6B). The weight of surviving larvae from St. Armand and St. Mathieu-de-Beloeil, QC was significantly greater on Cry1Fa vegetative tissue and not significantly different on reproductive tissue relative to non-Bt (Fig. 6C and D). On vegetative Cry1Fa tissue, 20, 49, and 17% of the St. Armand, QC larvae developed to first, second, and third instar during the 7-d bioassay, whereas 41, 35, and 5% reached these stages on non-Bt (Supplementary Table S4). For St. Mathieu-de-Beloeil, QC, 15, 54, and 33% of larvae reached the first, second, and third instar, respectively on Cry1Fa vegetative tissue while 31, 58, and 13% reached these stages on non-Bt (Supplementary Table S4). On reproductive Cry1Fa tissue, for St. Armand, 33, 60, and 1% and for St. Mathieu-de-Beloeil, QC, 27, 57, and 2% of larvae developed to the first, second, and third instar, respectively, whereas on non-Bt reproductive tissue, the proportion of larvae in each instar ranged from 43 to 56%, 37 to 50%, and 0 to 2% respectively (Supplementary Table S4). For comparison, 65 and 36% and 43 and 50% of the Delaware-SUS, ON strain developed to the first and second instar during the vegetative and reproductive tissue bioassays; no third instar larvae were observed (Supplementary Table S4). For Truro, NS, larval weight was not significantly different between Cry1Fa and non-Bt tissue regardless of plant stage (Fig. 6C and D). Surviving larvae from Truro, NS, on Cry1Fa vegetative tissue were 54, 51, and 4% first, second, and third instar and 63 and 31% were first and second instar on reproductive tissue, respectively. Of the larvae from Sussex, NB that survived on Cry1Fa vegetative tissue, 35, 81, and 0% were first, second, and third instar, respectively, compared to 45, 48, and 3% on non-Bt (Supplementary Table S4). On non-Bt reproductive tissue, 79 and 21% of surviving larvae from Truro, NS were first and second instar, respectively, at the end of the 7-d bioassay; and 63 and 31% were first and second instar on Cry1Fa (Supplementary Table S4).

**Fig. 7.**
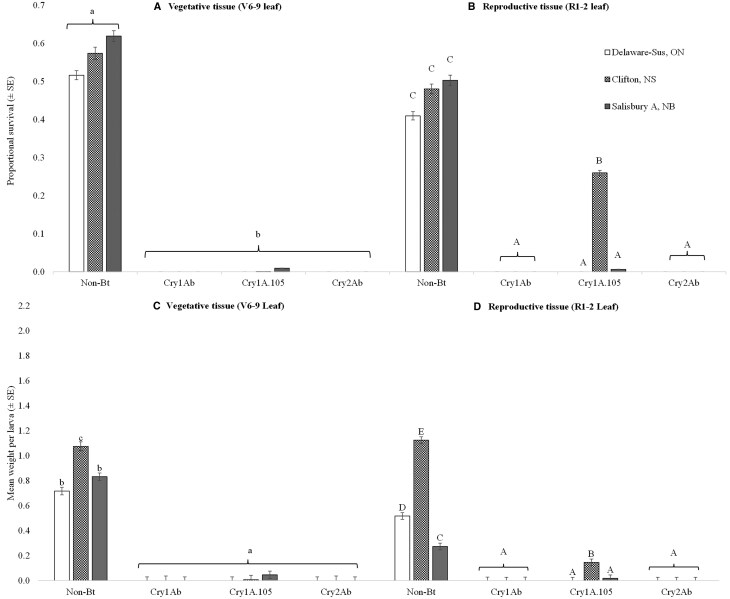
Proportional survival of *Ostrinia nubilalis* larvae from field-derived strains in Canada in 2023 after 7 d of exposure to A) vegetative or B) reproductive-stage leaf tissue and mean weight of surviving larvae on C) vegetative or D) reproductive-stage leaf tissue from non-Bt, Cry1Ab, Cry1A.105, or Cry2Ab corn. LSMEANS followed by the same letter within plant stage were not significantly different according to Tukey’s HSD test (*P *< 0.05).

For the 2023 field-derived strains, survival on Cry1Ab, Cry1A.105, and Cry2Ab vegetative tissue was nearly zero across strains and significantly lower than non-Bt (*F*_3, 451_ = 873.29, *P *< 0.0001) while survival on reproductive tissue varied by strain (Rep: *F*_6, 513_ = 28.45 *P *< 0.0001) (Fig. 7A and B). Larval weight differed by strain due to significant interactions (vegetative: *F*_6, 451_ = 7.40, *P *< 0.0001; reproductive: *F*_6, 513_ = 61.09, *P *< 0.0001) (Fig. 7C and D). Clifton, NS exhibited 26% survival on Cry1A.105 reproductive tissue; survivors were primarily first instars (one second instar) and significantly smaller than survivors on non-Bt (Fig. 7A and B, Supplementary Table S3). No survival was observed on Cry1Ab or Cry2Ab vegetative or reproductive leaf tissue (Fig. 7A and B).

**Fig. 8.**
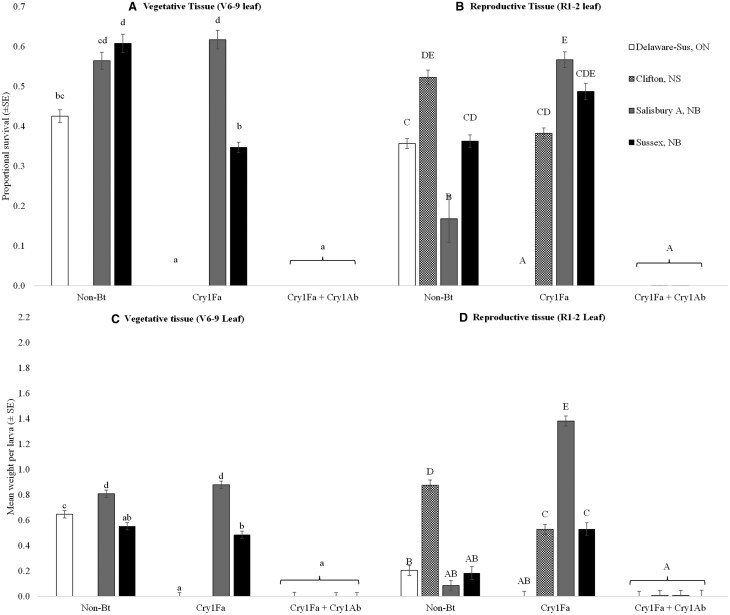
Proportional survival of *Ostrinia nubilalis* larvae from field-derived strains in Canada in 2023 after 7 d of exposure to A) vegetative or B) reproductive-stage leaf tissue and mean weight of surviving larvae C) vegetative or D) reproductive-stage leaf tissue from non-Bt, Cry1Fa, or Cry1Fa + Cry1Ab corn. LSMEANS followed by the same letter within plant stage were not significantly different according to Tukey’s HSD test (*P *< 0.05).

Survival of the 2023 strains on Cry1Fa, Cry1Fa + Cry1Ab, or non-Bt also varied by strain (vegetative: *F*_4, 368_ = 43.19, *P *< 0.0001; reproductive: *F*_6, 468_ = 50.67 *P *< 0.0001) (Fig. 8A and B). Salisbury A, NB showed similar survival on non-Bt and Cry1Fa vegetative tissue and greater survival on Cry1Fa reproductive tissue than non-Bt (Fig. 8A and B). Survival of Sussex, NB on Cry1Fa vegetative tissue was 47% of non-Bt; reproductive survival did not differ (Fig. 8A and B). No difference in survival was observed between non-Bt and Cry1Fa reproductive tissue for the Clifton, NS strain (Fig. 8B). No survival occurred on Cry1Fa + Cry1Ab except two Clifton, NS, larvae on reproductive tissue, which did not develop beyond first instars (Fig. 8B). Larval weight varied by strain (vegetative: *F*_4, 368_ = 69.31, *P *< 0.0001; reproductive: *F*_6, 468_=104.67, *P *< 0.0001) (Fig. 8C and D). Clifton, NS larvae were 20% smaller on Cry1Fa reproductive tissue relative to non-Bt and were comprised of 57 and 45% first and second instars, respectively, whereas 47, 35, and 19% reach first, second, and third instar on non-Bt, respectively (Fig. 8D, Supplementary Table S4). Conversely, Salisbury A and Sussex, NB larvae on Cry1Fa reproductive tissue were significantly larger (16.0- and 2.8-fold, respectively) than on non-Bt (Fig. 8D). For the NB strains, development was similar on Cry1Fa and non-Bt vegetative tissue and on Cry1Fa reproductive tissue, more developed to the second and third instar relative to non-Bt (Supplementary Table S4).

## Discussion

Our findings indicate that Cry1 toxin resistance is increasing in eastern Canada, with Cry1A.105 resistance emerging where Cry1Fa resistance was previously detected (Smith and Farhan 2023). In earlier work, 5 of 8 *O. nubilalis* strains collected in NS (2019 to 2020) were resistant to Cry1Fa (RR 15 to >41), four also showed elevated survival on Cry1A.105 (RR ≥ 2.3) and three on Cry1Ab (RR ≥ 2.5) (Smith and Farhan 2023). In this study, two strains from each province of NS (Truro and Clifton), NB (Sussex (2023) and Salisbury A), and QC (St. Armand and St. Mathieu-de-Beloeil) were resistant to both Cry1Fa and Cry1A.105 (RR >34 and >14, respectively) in diet-overlay bioassays. Tissue bioassays confirmed survival on Cry1Fa comparable to non-Bt, with some strains also showing elevated survival on Cry1A.105. Sentinel plot injury on Cry1Fa corn at Berwick, NS, Sussex, NB, St. Mathieu-de-Beloeil, QC, and Winchester, ON further supports increasing Cry1-resistance in eastern Canada. These results highlight the value of combining sentinel plots with bioassays to track Bt-resistance development and spread.

Cross-resistance among Cry1Fa and Cry1A.105 is plausible as both share a binding site in the midgut brush border membrane (BBM) of *O. nubilalis* (Hernández-Rodríguez et al. 2013). The Truro, NS strain was collected from a field planted with a grain corn hybrid producing Cry1Fa + Cry1Ab containing a 5% non-Bt integrated refuge. Diet-overlay bioassay results confirmed that this strain was resistant to Cry1Fa, Cry1A.105, and Cry1Ab. Tissue bioassays showed no survival difference between non-Bt and Cry1Fa; however, no survival was observed on Cry1Fa + Cry1Ab tissue. Similar results occurred for the other five strains with resistance to Cry1Fa and Cry1A.105, which also had significantly greater LC_50_ values for Cry1Ab relative to a susceptible strain. This result is consistent with a previous study that reported low survival of a Cry1Ab-resistant *O. nubilalis* strain on Cry1Fa tissue (Crespo et al. 2011). Although Cry1Fa and Cry1Ab share a binding site in *O. nubilalis*, they bind to additional unique sites in the BBM, which may lower the likelihood of cross-resistance between these two proteins as has recently been shown in the closely related species, the Asian corn borer, *O. furnacalis* (Lepidoptera: Crambidae) (Raeman et al. 2020, Wang et al. 2025).

The industry-led Agricultural Biotechnology Stewardship Technical Committee and CropLife Canada have proposed to the USEPA and CFIA that the threshold for unexpected injury (UXI) by *O. nubilalis* in Bt corn is reached when at least 5 cm of tunneling is observed in more than 2 of 30 (6.7%) Bt corn plants (CropLife 2020). This threshold was greatly exceeded in fields planted with Cry1Fa in NS in 2018 where high levels of Cry1Fa-resistance was confirmed (Smith et al. 2019). This threshold was exceeded at Sussex, NB in cultivars producing Cry1Fa, Cry1Ab, Cry1Ab + Vip3a, and Cry1A.105 + Cry2Ab in 2023. While the proposed threshold was not exceeded at St. Mathieu-de-Beloeil, QC and Winchester, ON sentinel sites, observed injury to Cry1Fa, Cry1Ab, and Cry1A.105 + Cry2Ab plants should be considered an early warning of resistance, corroborated by bioassay results.

Sentinel sites were initially established to monitor Bt resistance in *H. zea*; but increasing observations of *O. nubilalis* larvae and kernel injury at sites in NS, NB, QC, and eastern ON from 2020 to 2023 align with declining Bt susceptibility detected through diet-overlay bioassays (Smith and Farhan 2023). At Berwick, NS (46 to 100 km from original Cry1Fa-resistance detections), ear injury was observed on Cry1Fa corn in 2020, but not on Cry1Ab or Cry1A.105 + Cry2Ab hybrids. No injury was observed in 2021, yet Cry1Fa injury reappeared in 2022, possibly due to fluctuations in *O. nubilalis* population levels or phenology. NB sentinel monitoring began in 2021 (∼175 km west of NS detections); no injury was observed that year, but in 2022, minor injury was observed on Cry1Fa and Cry1Ab. Tissue assays showed elevated survival on Cry1Fa, while diet-overlay bioassays indicated significantly greater survival on Cry1Ab (RR 2.6) relative to a susceptible strain. By 2023, extensive stalk boring was observed on nearly all Bt and non-Bt plants, and bioassays confirmed resistance to Cry1Fa and Cry1A.105, suggesting a rapid increase in resistance allele frequencies within three years. Earlier low-level injury may have been missed, as 2023 was the first year of stalk sampling.

In Quebec, a 2019 strain from St. Mathieu-de-Beloeil exhibited Cry1Fa resistance (RR 35–41) and elevated survival on Cry1A.105 and Cry2Ab but remained susceptible to Cry1Ab (Smith and Farhan 2023). By 2022, strains from this site and St. Armand (∼80 km away) showed resistance to Cry1Fa and Cry1A.105 in diet-overlay and tissue bioassays, indicating regional spread and cross-resistance. Stalk sampling has not yet been conducted at QC sites; however, future monitoring is recommended, including in adjacent United States given St. Armand’s proximity to Vermont and New York. The Winchester, ON site (∼175 km southwest of the QC site) showed no evidence of Bt resistance before 2023. However, injury on Cry1Fa and Cry1Fa + Cry1Ab plants and low-level stalk boring suggest resistance alleles may now be present in eastern ON. A collection from this site did not produce a viable colony for bioassays. No evidence for resistance has been found in southwestern ON, but continued monitoring is critical to detect early spread into this high-production region, which could serve as a corridor to the U.S. Corn Belt.

Adult *O. nubilalis* dispersal likely contributes to spreading resistance. While most adults remain within 12 km of their natal field, ∼13% can disperse 50 to severalhundred kilometers within a generation with wind assistance (Palmer et al. 1985, Sappington 2018, 2024). The capacity for long-distance migration, coupled with high gene flow documented among North American populations, likely facilitates broad resistance allele movement (Krumm et al. 2008, Kim et al. 2009). Detection of the same genetic mutations in Cry1Fa-resistant *O. nubilalis* from Masstown, NS and Saint-Mathieu-de-Beloeil, QC populations, separated by >700 km, further supports the hypothesis that Bt resistance is spreading in eastern Canada (Farhan et al. 2023).

Several challenges have emerged in adapting the sentinel plot program for *O. nubilalis*. First, no commercialized sweet corn cultivars produce Cry1Fa because most ear-feeding lepidopteran pests are not susceptible to this toxin (Reay-Jones and Reisig 2014, Ostrem et al. 2016, Smith et al. 2017, Reay-Jones 2019). Consequently, field corn hybrids must be used to assess *O. nubilalis* Cry1Fa susceptibility. Second, single-toxin Bt hybrids have slowly been removed from the North American market, leaving few near-isoline cultivars for ideal comparators. Obtaining phenologically appropriate cultivars for short-season regions such as eastern Canada is also difficult due to limited availability. In this study, sweet corn sustained roughly twice as much *O. nubilalis* injury as field corn, possibly because it was more attractive or phenologically aligned for oviposition (Beard 1943, Revillon et al. 2024).

Another challenge is the decline in expertise and interest in monitoring and evaluating *O. nubilalis* injury following decades of successful Bt corn management, necessitating training for sentinel plot cooperators. Destructive stalk sampling, though labor intensive, is critical for detecting Bt-resistant *O. nubilalis* populations. The most extensive sampling occurred in 2023, revealing significant injury to Bt-corn in eastern Canada and the United States (Fisher et al. 2025). If stalk sampling of sentinel plots had been implemented earlier, perhaps earlier detection of this growing problem would have occurred. Given the vast distribution and host diversity of *O. nubilalis* in North America (Mason et al. 2018), combining sentinel plot sampling with collections from commercial fields for bioassays increases the likelihood of detecting Bt-resistance when *O. nubilalis* populations fluctuate annually.

For 25 yr, Bt susceptibility of *O. nubilalis* remained stable in North America (Siegfried and Hellmich 2012, Siegfried et al. 2014, Smith et al. 2019, Tabashnik et al. 2023). However, in 2018, NS field populations exhibited >30-fold Cry1Fa-resistance and extensive crop injury, meeting the definition of practical resistance where Bt efficacy is compromised and >50% of the population is resistant (Tabashnik et al. 2014, Smith et al. 2019). With resistance now emerging in Canada and the United States, strengthening resistance management strategies is critical to slow its spread and protect yield and quality. Mitigation should begin wherever Bt corn injury is documented, regardless of UXI thresholds, including Atlantic Canada and QC. Recommended measures include increasing non-Bt refuge areas (Tabashnik and Carrière 2019), destroying overwintering *O. nubilalis* habitat mechanically (Schaafsma et al. 1996), and considering chemical and biological controls (Kaçar et al. 2023). Bt corn remains a cornerstone of *O. nubilalis* management throughout North America, but recent findings suggest that we may be entering an era of resistance to current Bt toxins.

We recommend implementing a sentinel plot program for Bt-resistance monitoring across the North American range of *O. nubilalis*. This approach would complement existing laboratory bioassays, provide field-relevant susceptibility data, and enhance collection efforts in low-population regions. Sentinel plots could provide early warnings of resistance evolution or spread and improve mitigation response times. Success will require collaboration between public researchers and industry partners to supply Bt and non-Bt corn lines of appropriate maturity for diverse regions throughout the range of this pest. Now is the time to reinvigorate grower education on integrated pest management and resistance management strategies for *O. nubilalis* while expanding resistance monitoring efforts.

## Supplementary Material

toag077_Supplementary_Data
