# Supplementary material for: Sentinel monitoring for resistance to Bt toxins in European corn borer (Lepidoptera: Crambidae) in Canada
Source: J Econ Entomol. 2026 Apr 23;119(3):2224–40. doi: 10.1093/jee/toag077 (PMC13268522; doi:10.1093/jee/toag077)
Supplement: toag077_Supplementary_Data [file toag077_supplementary_data.zip › Supplemental Table 1.docx]

**Supplemental Table 1.** Planting and sampling dates of sentinel sites for monitoring injury by *Ostrinia nubilalis* to non-Bt and Bt corn in Canada, 2019-2023.

| Year^a, b^ | | Site | Planting date | Plot size | | Ear sampling date (Stage) | | Stalk sampling date (Stage) | |
| --- | --- | --- | --- | --- | --- | --- | --- | --- | --- |
|  |  |  |  | No. rows | Length (m) | Sweet corn | Field corn | Sweet corn | Field corn |
| 2019 | Ridgetown, ON | | Early – 12 Jun | 4 | 40 | 24 Sept (R6) | - | - | - |
|  |  | | Late – 26 Jun | 4 | 40 | 24 Sept (R4) | - | - | - |
| 2020^c^ | Ridgetown, ON | | Early – 9 Jun | 4 | 40 | 23 Sept (R6) | - | - | - |
|  |  |  | Late – 26 Jun | 4 | 40 | 23 Sept (R4) | - | - | - |
| Winchester, ON | | | 02 Jun | 12 | 20 | 2, 14 Sept (R4) | - | - | - |
| St. Mathieu-de-Beloeil, QC | | | 08 Jun | 4 | 30 | 22 Sept (R5) | - | - | - |
| Berwick, NS | | | 14 Jun | 4 | 30 | 26 Aug (R3) | - | - | - |
| 2021^c^ | Ridgetown, ON | | Early – 1 Jun | 4 | 40 | 27 Aug (R4) | - | - | - |
|  |  |  | Late – 26 Jun | 4 | 40 | 28 Sept (R6) | - | - | - |
| Winchester, ON | | | 21 May | 12 | 20 | 23 Aug (R3) | - | - | - |
| St. Mathieu-de-Beloeil, QC | | | 07 Jun | 4 | 30 | 31 Aug (R6) | - |  |  |
| Cambridge, NS | | | Early – 07 Jun | 4 | 30 | 8 Sept (R4) | - | - | - |
|  |  |  | Late – 21 Jun | 4 | 30 | 29 Sept (R5) | - | - | - |
| Sussex, NB | | | 16 Jun | 4 | 30 | 24 Sept (R4) | - | - | - |
| Freetown, PEI | | | 6 Jul | 4 | 20 | 1 Oct (R5) |  |  |  |
| 2022^d^ | Ridgetown, ON | | Early – 14 Jun | 4 | 40 | 20 Sep (R6) | 20 Sep (R6) | - | - |
|  |  |  | Late – 7 Jul | 4 | 40 | 23 Sep (R3) | 23 Sep (R3) |  |  |
| Winchester, ON | | | 07 Jun | 12 | 15 | 30 Aug (R4) | 30 Aug (R4) | - | - |
| St. Mathieu-de-Beloeil, QC | | | 25 May (Grain) | 4 | 30 | 19 Sept (R3) | 19 Sept (R4) | - | - |
|  |  |  | 7 Jun (Sweet) | 4 | 30 |  |  | - | - |
| Cambridge, NS | | | 29 Jun | 4 | 30 | 28 Sept (R5) | - | - | - |
| Sussex, NB | | | 07 Jun | 4 | 30 | 28 Sept (R4) | 28 Sept (R4) | - | - |
| 2023^d^ | Ridgetown, ON | | Early – 5 Jun | 4 | 40 | 18 Sept (R5) | 19 Sept (R6) | - | - |
|  |  |  | Late – 12 Jul | 4 | 40 | 13 Oct (R6) | 20 Oct (R5) | 24 Oct (R6) | 24 Oct (R6) |
| Winchester, ON | | | Early – 8 Jun | 15 | 20 | 18 Sept (R5) | 19 Sept (R5) | 20 Nov (R6) | 20 Nov (R6) |
|  |  |  | Late – 12 Jul | 15 | 20 | 16 Oct (R6) | 20 Oct (R5) | 20 Nov (R6) | 20 Nov (R6) |
| St. Mathieu-de-Beloeil, QC | | | 09 Jun | 4 | 30 | 18 Sept (R3) | 18 Sept (R4) | - | - |
| Sussex, NB | | | 30 May | 4 | 30 | 22 Sept (R4) | 22 Sept (R4) | 26 Sept (R4) | 26 Sept (R4) |

^a^ Sweet corn cultivars planted in all years: 1) ‘Attribute BC0805’ producing Cry1Ab, 2) ‘Attribute II Remedy’ producing Cry1Ab and Vip3A, and 3) their non-Bt isoline ‘Providence’ (Syngenta Seeds); 4) ‘Performance Series Obsession II’ producing Cry1A.105 + Cry2Ab, and 5) its non-Bt isoline ‘Obsession I’ (Bayer-Seminis Seeds).

^b^ All plots were planted with pure stands of each cultivar, i.e. without integrated non-Bt refuge.

^c^ In 2020 and 2021, plots of a commercially available grain corn hybrid Hybrid D (Cry1Fa; 72 comparative relative maturity (CRM)) (Pioneer Hi-Bred Production Co. Chatham, ON) were added to the sentinel sites.

^d^ In 2022 and 2023, plots of grain corn cultivars Hybrid B (Cry1Fa; 103 CRM), Hybrid C (Cry1Fa and Cry1Ab; 82 CRM), and Hybrid A (non-Bt; 80 CRM) (Pioneer Hi-Bred Production Co. Chatham, ON) were added at all sentinel sites except Cambridge, NS.
