# Supplementary material for: Sentinel monitoring for resistance to Bt toxins in European corn borer (Lepidoptera: Crambidae) in Canada
Source: J Econ Entomol. 2026 Apr 23;119(3):2224–40. doi: 10.1093/jee/toag077 (PMC13268522; doi:10.1093/jee/toag077)
Supplement: toag077_Supplementary_Data [file toag077_supplementary_data.zip › Supplemental Table 2.docx]

**Supplemental Table 2.** Incidence of ears with larvae and injury by Ostrinia nubilalis observed in assessments of sentinel plots of sweet and grain corn in Canada from 2019-2023.

| Year | Planting date | Proportion of plants with ear injury by *Ostrinia nubilalis* (n^a^) **Number of larvae observed** | | | | | | | | |  |
| --- | --- | --- | --- | --- | --- | --- | --- | --- | --- | --- | --- |
|  |  | Providence  (Non-Bt) | Attribute  (Cry1Ab) | Remedy (Cry1Ab + Vip3A) | Obsession I  (Non-Bt) | Obsession II  (Cry1A.105 + Cry2Ab) | Hybrid A  (Non-Bt) | Hybrid B (Cry1Fa) | Hybrid D (Cry1Fa) | Hybrid (C)  (Cry1Fa + Cry1Ab) |  |
| Berwick (2020) and Cambridge (2021-2022), Nova Scotia | | | | | | | | | | | |
| 2020 | 14 Jun | 0.12 (50) **4** | 0 (50) | 0 (50) | 0 (50) **6** | 0 (50) | . | 0.16 (50) **6** | . | . |  |
| 2021 | 7 Jun | 0 (50) | 0 (50) | 0 (50) | 0 (50) | 0 (50) | . | 0 (50) | . | . |  |
|  | 21 Jun | 0 (50) | 0 (50) | 0 (50) | 0 (50) | 0 (50) | . | 0 (50) | . | . |  |
| 2022 | 29 Jun | 0 (50) | 0 (50) | 0 (50) | 0.02 (50) **1** | 0 (50) | 0.02 (50) | . | 0.02 (50) | . |  |
| Sussex, New Brunswick | | | | | | | | | | | |
| 2021 | 16 Jun | 0 (25) | 0 (25) **1** | 0 (25) | 0 (25) | 0 (25) | . | 0 (25) | . | . |  |
| 2022 | 7 Jun | 0 (50) | 0.03 (100) **1** | 0 (200) | 0 (50) | 0 (100) | 0 (50) | . | 0.01 (100) | 0 (100) |  |
| 2023 | 30 May | .^b^ | 0 (25) | 0 (25) | 0.1 (10) **1** | 0 (25) | 0.04 (25) **4** | . | 0 (25) | 0 (25) |  |
| Freetown, Prince Edward Island | | | | | | | | | | |  |
| 2021 | 6 Jul | 0 (50) | 0(50) | 0(50) | 0(50) | 0(50) | . | 0(50) | . | . |  |
| St. Mathieu-de-Beloeil, Québec | | | | | | | | | | | |
| 2020 | 8 Jun | 0 (50) | 0 (100) | 0 (100) | 0 (50) **1** | 0 (50) | . | 0 (50) **1** | . | . |  |
| 2021 | 7 Jun | 0 (100) | 0 (100) | 0 (100) | 0 (100) | 0 (100) | . | 0 (100) | . | . |  |
| 2022 | 25 May | 0.2 (25) **4** | 0.12 (25) **3** | 0 (25) | 0.24 (25) **8** | 0 (25) | 0 (25) | . | 0.32 (25) **12** | 0 (25) |  |
| 2023 | 9 Jun | 0 (50) | 0 (100) | 0 (100) | 0 (50) | 0 (100) | 0 (100) | . | 0 (100) | 0 (100) |  |
| Winchester, Ontario | | | | | | | | | | | |
| 2020 | 2 Jun | 0.08 (130) **21** | 0 (110) | 0 (110) | 0 (130) **8** | 0 (110) | . | . | . | . |  |
| 2021 | 21 May | 0.16 (100) **37** | 0.01 (100) **1** | 0 (100) | 0.18 (100) **38** | 0 (100) | . | . | . | . |  |
| 2022 | 7 Jun | 0.24 (50) **14** | 0 (100) | 0 (100) | 0.22 (50) **13** | 0 (100) | 0.01 (100) **1** | . | 0 (100) | 0 (100) |  |
| 2023 | 8 Jun | 0.12 (50) **6** | 0 (50) | 0 (50) | 0.3 (50) **19** | 0 (50) | 0.17 (50) **12** | 0.02 (50) | 0.04 (50) | 0.14 (50) |  |
|  | 12 Jul | 0.02 (50) | 0 (50) | 0 (50) | 0.02 (50) | . | . | 0.02 (50) | . | 0.08 (50) |  |
| Ridgetown, Ontario | | | | | | | | | | | |
| 2019 | 12 Jun | 0 (50) | 0 (100) | 0 (100) | 0 (50) | 0 (100) | . | 0 (100) | . | . |  |
|  | 26 Jun | 0 (50) | 0 (100) | 0 (100) | 0 (50) | 0 (100) | . | 0 (100) | . | . |  |
| 2020 | 9 Jun | 0 (50) | 0 (100) | 0 (100) | 0 (50) | 0 (100) | . | 0 (50) | . | . |  |
|  | 26 Jun | 0 (50) | 0 (100) | 0 (100) | 0.02 (50) **1** | 0 (100) | . | . | . | . |  |
| 2021 | 1 Jun | 0 (50) | 0 (50) | 0 (50) | 0 (50) | 0 (50) | . | 0 (50) | . | . |  |
|  | 26 Jun | 0 (100) | 0 (100) | 0 (100) | 0 (100) | 0 (100) | . | 0 (100) | . | . |  |
| 2022 | 14 Jun | 0 (50) | 0 (50) | 0 (50) | 0 (50) | 0 (50) | . | 0 (50) | . | . |  |
|  | 7 Jul | . | . | . | . | . | . | . | . | . |  |
| 2023 | 5 Jun | 0 (50) | 0 (100) | 0 (100) | 0 (50) | 0 (100) | 0 (50) | 0 (100) | . | 0 (100) |  |
|  | 12 Jul | 0 (50) | 0 (100) | 0 (100) | 0 (50) | 0 (100) | 0 (50) | 0 (100) | . | 0 (100) |  |

^a^ Number of plants sampled within the plot.

^b^ Plot was destroyed by *O. nubilalis* injury resulting in poor ear production and lodging; therefore, ear injury could not be assessed.
