# Supplementary material for: Sentinel monitoring for resistance to Bt toxins in European corn borer (Lepidoptera: Crambidae) in Canada
Source: J Econ Entomol. 2026 Apr 23;119(3):2224–40. doi: 10.1093/jee/toag077 (PMC13268522; doi:10.1093/jee/toag077)
Supplement: toag077_Supplementary_Data [file toag077_supplementary_data.zip › Supplemental Table 3.docx]

**Supplemental Table 3.** The distribution of first, second, and third instar Ostrinia nubilalis larvae surviving exposure to vegetative or reproductive-stage leaf tissue from non-Bt, Cry1Ab, Cry1A.105, or Cry2Ab plants in a 7-day bioassay.

| **Year of Collection**  Field-derived strain |  | Proportion of larvae (mean ± SE) | | | | | |
| --- | --- | --- | --- | --- | --- | --- | --- |
|  |  | Vegetative tissue (V6-9 leaf) | | | Reproductive tissue (R1-2 leaf) | | |
| **2022** | Bt toxin | 1^st^ instar | 2^nd^ instar | 3^rd^ instar | 1^st^ instar | 2^nd^ instar | 3^rd^ instar |
| Delaware-Sus, ON | Non-Bt | 0.69 ± 0.058 | 0.28 ± 0.068 | 0.0 | 0.58 ± 0.052 | 0.41 ± 0.051 | 0.0 |
|  | Cry1Ab | 0.0 | 0.0 | 0.0 | 0.0 | 0.0 | 0.0 |
|  | Cry1A.105 | 0.0 | 0.0 | 0.0 | 0.0 | 0.0 | 0.0 |
|  | Cry2Ab2 | 0.0 | 0.0 | 0.0 | 0.0 | 0.0 | 0.0 |
| St. Armand, QC | Non-Bt | 0.61 ± 0.159 | 0.49 ± 0.064 | 0.0 | 0.49 ± 0.060 | 0.44 ± 0.058 | 0.07 ± 0.035 |
|  | Cry1Ab | 0.0 | 0.0 | 0.0 | 0.0 | 0.0 | 0.0 |
|  | Cry1A.105 | 1.00 ± 0.000 | 0.0 | 0.0 | 0.50 ± 0.050 | 0.50 ± 0.050 | 0.0 |
|  | Cry2Ab2 | 0.0 | 0.0 | 0.0 | 0.0 | 0.0 | 0.0 |
| St. Mathieu-de-Beloeil, QC | Non-Bt | 0.76 ± 0.107 | 0.33 ± 0.046 | 0.0 | . | . | . |
|  | Cry1Ab | 0.0 | 0.0 | 0.0 | . | . | . |
|  | Cry1A.105 | 0.97 ± 0.028 | 0.03 ± 0.028 | 0.0 | . | . | . |
|  | Cry2Ab2 | 0.0 | 0.0 | 0.0 | . | . | . |
| St. Georges, PEI | Non-Bt | . | . | . | 1.00 ± 0.000 | 0.0 | 0.0 |
|  | Cry1Ab | . | . | . | 0.0 | 0.0 | 0.0 |
|  | Cry1A.105 | . | . | . | 0.0 | 0.0 | 0.0 |
|  | Cry2Ab2 | . | . | . | 0.0 | 0.0 | 0.0 |
| Carman, MB | Non-Bt | 0.51 ± 0.087 | 0.44 ± 0.086 | 0.0 | . | . | . |
|  | Cry1Ab | 1.00 ± 0.000 | 0.0 | 0.0 | . | . | . |
|  | Cry1A.105 | 0.0 | 0.0 | 0.0 | . | . | . |
|  | Cry2Ab2 | 0.0 | 0.0 | 0.0 | . | . | . |
| **2023** |  |  |  |  |  |  |  |
| Delaware-Sus, ON | Non-Bt | 0.52 ± 0.070 | 0.38 ± 0.062 | 0.09 ± 0.034 | 0.63 ± 0.056 | 0.39 ± 0.056 | 0.0 |
|  | Cry1Ab | 0.0 | 0.0 | 0.0 | 0.0 | 0.0 | 0.0 |
|  | Cry1A.105 | 0.0 | 0.0 | 0.0 | 0.0 | 0.0 | 0.0 |
|  | Cry2Ab2 | 0.0 | 0.0 | 0.0 | 0.0 | 0.0 | 0.0 |
| Clifton, NS | Non-Bt | 0.19 ± 0.049 | 0.66 ± 0.059 | 0.14 ± 0.048 | 0.18 ± 0.047 | 0.58 ± 0.057 | 0.25 ± 0.051 |
|  | Cry1Ab | 0.0 | 0.0 | 0.0 | 0.0 | 0.0 | 0.0 |
|  | Cry1A.105 | 1.00 ± 0.000 | 0.0 | 0.0 | 0.99 ± 0.013 | 0.01 ± 0.013 | 0.0 |
|  | Cry2Ab2 | 0.0 | 0.0 | 0.0 | 0.0 | 0.0 | 0.0 |
| Salisbury A, NS | Non-Bt | 0.34 ± 0.042 | 0.52 ± 0.047 | 0.24 ± 0.046 | 1.00 ± 0.000 | 0.0 | 0.0 |
|  | Cry1Ab | 0.0 | 0.0 | 0.0 | 0.0 | 0.0 | 0.0 |
|  | Cry1A.105 | 0.78 ± 0.165 | 0.22 ± 0.047 | 0.24 ± 0.046 | 0.92 ± 0.029 | 0.08 ± 0.029 | 0.0 |
|  | Cry2Ab2 | 0.0 | 0.0 | 0.0 | 0.0 | 0.0 | 0.0 |
