# Supplementary material for: Sentinel monitoring for resistance to Bt toxins in European corn borer (Lepidoptera: Crambidae) in Canada
Source: J Econ Entomol. 2026 Apr 23;119(3):2224–40. doi: 10.1093/jee/toag077 (PMC13268522; doi:10.1093/jee/toag077)
Supplement: toag077_Supplementary_Data [file toag077_supplementary_data.zip › Supplemental Table 4.docx]

**Supplemental Table 4.** The distribution of first, second, and third instar Ostrinia nubilalis larvae surviving exposure to vegetative or reproductive-stage leaf tissue from non-Bt, Cry1Fa, or Cry1Fa + Cry1Ab plants in a 7-day bioassay.

| **Year of collection** |  | Proportion of larvae (mean ± SE) | | | | | |
| --- | --- | --- | --- | --- | --- | --- | --- |
| Field-derived strain |  | Vegetative tissue (V6-9 leaf) | | | Reproductive tissue (R1-2 leaf) | | |
| **2022** |  | 1^st^ instar | 2^nd^ instar | 3^rd^ instar | 1^st^ instar | 2^nd^ instar | 3^rd^ instar |
| Delaware-Sus, ON | Non-Bt | 0.65 ± 0.060 | 0.36 ± 0.060 | 0.0 | 0.43 ± 0.094 | 0.50 ± 0.093 | 0.0 |
|  | Cry1Fa | 1.00 ± 0.00 | 0.0 | 0.0 | 0.0 | 0.0 | 0.0 |
|  | Cry1Fa + Cry1Ab | 0.0 | 0.0 | 0.0 | 0.0 | 0.0 | 0.0 |
| St. Armand, QC | Non-Bt | 0.41 ± 0.078 | 0.35 ± 0.055 | 0.05 ± 0.017 | 0.56 ± 0.067 | 0.37 ± 0.065 | 0.02 ± 0.023 |
|  | Cry1Fa | 0.20 ± 0.047 | 0.49 ± 0.046 | 0.17 ± 0.033 | 0.33 ± 0.054 | 0.60 ± 0.052 | 0.01 ± 0.008 |
|  | Cry1Fa + Cry1Ab | 1.00 ± 0.00 | 0.0 | 0.0 | 0.0 | 0.0 | 0.0 |
| St. Mathieu-de-Beloeil, QC | Non-Bt | 0.31 ± 0.043 | 0.58 ± 0.046 | 0.13 ± 0.044 | 0.43 ± 0.058 | 0.54 ± 0.059 | 0.0 |
|  | Cry1Fa | 0.15 ± 0.045 | 0.54 ± 0.052 | 0.33 ± 0.050 | 0.27 ± 0.058 | 0.57 ± 0.063 | 0.02 ± 0.014 |
|  | Cry1Fa + Cry1Ab | 0.0 | 0.0 | 0.0 | 0.0 | 0.0 | 0.0 |
| Truro, NS | Non-Bt | 0.62 ± 0.050 | 0.36 ± 0.048 | 0.01 ± 0.010 | 0.79 ± 0.065 | 0.21 ± 0.065 | 0.0 |
|  | Cry1Fa | 0.54 ± 0.005 | 0.51 ± 0.051 | 0.04 ± 0.019 | 0.63 ± 0.077 | 0.31 ± 0.065 | 0.0 |
|  | Cry1Fa + Cry1Ab | 0.0 | 0.0 | 0.0 | 0.0 | 0.0 | 0.0 |
| Sussex, NB | Non-Bt | 0.45 ± 0.049 | 0.48 ± 0.051 | 0.03 ± 0.023 | 0.98 ± 0.367 | 0.02 ± 0.011 | 0.0 |
|  | Cry1Fa | 0.35 ± 0.173 | 0.81 ± 0.106 | 0.0 | 0.75 ± 0.250 | 0.25 ± 0.250 | 0.0 |
|  | Cry1Fa + Cry1Ab | 0.0 | 0.0 | 0.0 | 0.0 | 0.0 | 0.0 |
| St. Georges, PEI | Non-Bt | 0.70 ± 0.049 | 0.30 ± 0.173 | 0.0 | 0.59 ± 0.049 | 0.36 ± 0.050 | 0.0 |
|  | Cry1Fa | 0.0 | 0.0 | 0.0 | 0.0 | 0.0 | 0.0 |
|  | Cry1Fa + Cry1Ab | 0.0 | 0.0 | 0.0 | 0.79 ± 0.077 | 0.03 ± 0.028 | 0.0 |
| Carman, MB | Non-Bt | 0.98 ± 0.023 | 0.05 ± 0.045 | 0.0 | 0.64 ± 0.069 | 0.35 ± 0.069 | 0.0 |
|  | Cry1Fa | 0.0 | 0.0 | 0.0 | 0.0 | 0.0 | 0.0 |
|  | Cry1Fa + Cry1Ab | 0.0 | 0.0 | 0.0 | 0.0 | 0.0 | 0.0 |
| **2023** |  |  |  |  |  |  |  |
| Delaware-Sus, ON | Non-Bt | 0.67 ± 0.051 | 0.31 ± 0.049 | 0.0 | 0.97 ± 0.020 | 0.01 ± 0.012 | 0.0 |
|  | Cry1Fa | 0.0 | 0.0 | 0.0 | 0.0 | 0.0 | 0.0 |
|  | Cry1Fa + Cry1Ab | 0.0 | 0.0 | 0.0 | 0.0 | 0.0 | 0.0 |
| Clifton, NS | Non-Bt | . | . | . | 0.47 ± 0.061 | 0.35 ± 0.050 | 0.19 ± 0.047 |
|  | Cry1Fa | . | . | . | 0.57 ± 0.065 | 0.45 ± 0.067 | 0.0 |
|  | Cry1Fa + Cry1Ab | . | . | . | 1.00 ± 0.000 | 0.0 | 0.0 |
| Salisbury A, NB | Non-Bt | 0.51 ± 0.047 | 0.42 ± 0.044 | 0.06 ± 0.027 | 0.95 ± 0.026 | 0.03 ± 0.022 | 0.02 ± 0.016 |
|  | Cry1Fa | 0.43 ± 0.054 | 0.51 ± 0.053 | 0.06 ± 0.026 | 0.10 ± 0.026 | 0.57 ± 0.051 | 0.33 ± 0.050 |
|  | Cry1Fa + Cry1Ab | 0.0 | 0.0 | 0.0 | 1.00 ± 0.000 | 0.0 | 0.0 |
| Sussex, NB | Non-Bt | 0.81 ± 0.039 | 0.18 ± 0.037 | 0.0 | 0.89 ± 0.048 | 0.06 ± 0.030 | 0.0 |
|  | Cry1Fa | 0.69 ± 0.064 | 0.28 ± 0.057 | 0.01 ± 0.014 | 0.60 ± 0.074 | 0.40 ± 0.074 | 0.0 |
|  | Cry1Fa + Cry1Ab | 0.0 | 0.0 | 0.0 | 0.0 | 0.0 | 0.0 |
